# New reference genome sequences of hot pepper reveal the massive evolution of plant disease-resistance genes by retroduplication

**DOI:** 10.1186/s13059-017-1341-9

**Published:** 2017-11-01

**Authors:** Seungill Kim, Jieun Park, Seon-In Yeom, Yong-Min Kim, Eunyoung Seo, Ki-Tae Kim, Myung-Shin Kim, Je Min Lee, Kyeongchae Cheong, Ho-Sub Shin, Saet-Byul Kim, Koeun Han, Jundae Lee, Minkyu Park, Hyun-Ah Lee, Hye-Young Lee, Youngsill Lee, Soohyun Oh, Joo Hyun Lee, Eunhye Choi, Eunbi Choi, So Eui Lee, Jongbum Jeon, Hyunbin Kim, Gobong Choi, Hyeunjeong Song, JunKi Lee, Sang-Choon Lee, Jin-Kyung Kwon, Hea-Young Lee, Namjin Koo, Yunji Hong, Ryan W. Kim, Won-Hee Kang, Jin Hoe Huh, Byoung-Cheorl Kang, Tae-Jin Yang, Yong-Hwan Lee, Jeffrey L. Bennetzen, Doil Choi

**Affiliations:** 1 Department of Plant Science, Plant Genomics and Breeding Institute, Research Institute for Agriculture and Life Sciences, Seoul National University, Seoul, 08826 South Korea; 2 Interdisciplinary Program in Agricultural Genomics, Seoul National University, Seoul, 08826 South Korea; 3 Department of Agricultural Plant Science, Institute of Agriculture & Life Science, Gyeongsang National University, Jinju, 52828 South Korea; 4 Korean Bioinformation Center, Korea Research Institute of Bioscience and Biotechnology, Daejon, 34141 South Korea; 5 Department of Agricultural Biotechnology, Seoul National University, Seoul, 08826 South Korea; 6 Department of Horticultural Science, Kyungpook National University, Daegu, 41566 South Korea; 7 Vegetable Breeding Research Center, Seoul National University, Seoul, 08826 South Korea; 8 Department of Horticulture, Chonbuk National University, Jeonju, 54896 South Korea; 9 Department of Genetics, University of Georgia, Athens, GA 30602-7223 USA

**Keywords:** NLR, Retroduplication, LTR-retrotransposon, Disease-resistance gene, Genome evolution

## Abstract

**Background:**

Transposable elements are major evolutionary forces which can cause new genome structure and species diversification. The role of transposable elements in the expansion of nucleotide-binding and leucine-rich-repeat proteins (NLRs), the major disease-resistance gene families, has been unexplored in plants.

**Results:**

We report two high-quality de novo genomes (*Capsicum baccatum* and *C. chinense*) and an improved reference genome (*C. annuum*) for peppers. Dynamic genome rearrangements involving translocations among chromosomes 3, 5, and 9 were detected in comparison between *C. baccatum* and the two other peppers. The amplification of *athila* LTR-retrotransposons, members of the *gypsy* superfamily, led to genome expansion in *C. baccatum*. In-depth genome-wide comparison of genes and repeats unveiled that the copy numbers of NLRs were greatly increased by LTR-retrotransposon-mediated retroduplication. Moreover, retroduplicated NLRs are abundant across the angiosperms and, in most cases, are lineage-specific.

**Conclusions:**

Our study reveals that retroduplication has played key roles for the massive emergence of NLR genes including functional disease-resistance genes in pepper plants.

**Electronic supplementary material:**

The online version of this article (doi:10.1186/s13059-017-1341-9) contains supplementary material, which is available to authorized users.

## Background

Long terminal repeat retrotransposons (LTR-Rs) are a major evolutionary force in animals, fungi, and, especially, plants. They comprise > 75% of many plant genomes and cause genomic instability, including genome expansion by amplification using an RNA intermediate [1]. Besides genome expansion, LTR-Rs facilitate the creation of new candidate genes called retrogenes by means of retroduplication, in which spliced messenger RNA is captured, reverse transcribed, and subsequently integrated into the genome by a retrotransposon [2–4]. In contrast to transduplication caused by class II transposable elements (TEs) [5, 6], the distinctive features of retrogenes are: (1) intron loss compared to their parental source genes; (2) the presence of a 3′ poly(A) tail; and (3) flanking direct repeats [7].

The evolutionary forces acting on most plant retrogenes are still largely unclear [3, 8–11]. Although LTR-Rs are the most abundant TEs in all but the tiniest plant genomes, few studies have been reported on the detection of retrogenes generated by LTR-Rs in plants [12, 13]. Wang et al. [3] identified 27 retrogenes within LTR-Rs in rice and concluded that retrogenes that originated within LTR-Rs were often not classified as retrogenes, partly because of the rapid destruction of the LTR-R structure by illegitimate recombination [14]. Moreover, they suggested that the retrogenes might be very frequent in grass species due to the abundance of LTR-Rs. In agreement with this prediction, recent studies have reported the genome-wide identification of hundreds of retrogenes within LTR-Rs in maize [15], rice, and sorghum [16] as well as the existence of retrogenes captured by LTR-Rs in *Arabidopsis* [4]. However, most of those retrogenes were classified as pseudogenes or uncharacterized genes.

Previous studies reported the massive capture of specific gene families by certain TEs and suggested a correlation between TE-mediated gene duplication and specific gene family expansion [17, 18]. The nucleotide-binding and leucine-rich-repeat proteins (NLRs) represent a highly amplified gene family in plants and provide the majority of functional plant disease resistance loci [19–21]. Comparative genomic analyses have suggested the possibility of LTR-Rs and NLRs co-evolution, partly because they are often co-localized [20, 22, 23]. Because the NLRs usually reside in clusters within genomes, NLR expansions have been mainly interpreted as the products of ectopic recombinational duplications [19, 24].

Here, we report high-quality de novo genome sequences of two domesticated *Capsicum* species and also improved the quality of the reference pepper genome [25]. Comparative analyses of the three pepper genomes, with other plant genomes as outgroups, provided insights into genome evolution and species diversification in the genus *Capsicum*. Our analyses unveil an important mechanism for the massive emergence of new plant NLRs by LTR-R-mediated retroduplication and show the dynamic evolutionary processes for functional disease resistance genes in plants.

## Results and discussion

### De novo sequencing, assembly, and annotation of *Capsicum* genomes

We sequenced and assembled the genome sequences of *Capsicum baccatum* PBC81 (hereafter, Baccatum) and *C. chinense* PI159236 (hereafter, Chinense) using Illumina HiSeq 2500 with library insert sizes in the range of 200 bp–10 kb (Additional file 1: Table S1-S3). The estimated genome sizes of Baccatum and Chinense, based on 19-mer analysis, were 3.9 and 3.2 Gb, respectively (Additional file 1: Figure S1). The assembled genomes of Baccatum and Chinense constituted 3.2 and 3.0 Gb (83% and 94% of the estimated genome sizes, respectively) and had scaffold (contig) N50 sizes of 2.0 Mb (39 kb) and 3.3 Mb (50 kb), respectively (Additional file 1: Table S3). We annotated the protein-coding genes in the Baccatum and Chinense assemblies as well as those in the pre-existing *C. annuum* CM334 genome [25] (hereafter, Annuum) for detailed comparative analysis (Additional file 1: Figure S2). On average, ~ 35,000 genes were annotated in each species (Additional file 1: Table S4). Our analysis revealed higher gene coverage in the updated gene model than that in the previous gene model of Annuum (Additional file 1: Table S5). Furthermore, a comparison of the updated and previous gene models of Annuum revealed ~ 10,000 genes that did not overlap between the two gene models, suggesting that most of the non-overlapping genes in the previous version were associated with TEs (Additional file 1: Figure S3).

A high-density genetic map of each species was generated by genotyping-by-sequencing on F2 populations [26, 27]. After breaking up chimeric scaffolds on the basis of genetic map information, we organized the assembled genome sequences into 12 chromosomes-scale pseudomolecules. Overall, 87% of Baccatum (2.8 Gb in 2083 scaffolds) and 89% of Chinense (2.8 Gb in 1,557 scaffolds) in assembled genomes were ordered by the genetic map and inspected for syntenic inferences with the updated pseudomolecules of Annuum (Additional file 1: Table S6). We validated the assembled genome sequences by reference guided mapping using the refined single-end and paired-end data, and alignment to the de novo transcriptome assembly of each species (Additional file 1: Table S7 and S8). In total, we detected more than 98.1% of the filtered raw sequences (>98% identity) and more than 93.4% of the assembled transcriptomes (> 98% identity and 80% of query coverage) in the genome assemblies (Additional file 1: Table S8). Taken together, our analyses provide the de novo reference genome sequences of two new pepper species as well as an improved Annuum genome sequences.

Repeat annotation was performed with the assembled genomes and the initial contigs covering the estimated genome sizes of the three species (Additional file 1: Figure S4, Table S9 and S10). Overall, ~ 85% of the initial contigs were annotated as repeat sequences. LTR-Rs of the Ty3-*gypsy* superfamily accounted for about half of the entire genome in each of the three species (Additional file 1: Table S9 and S10). Among the subgroups of the *gypsy* superfamily, *del* elements comprised the largest fraction, representing 41.5%, 34.9%, and 41.7% (1482, 1337, and 1,343 Mb) in Annuum, Baccatum and Chinense, respectively. Furthermore, *athila* elements were more abundant (> 2-fold) in Baccatum, indicating that the *athila* subgroup contributed to species-specific genome expansion in the Baccatum lineage (Additional file 1: Table S10).

### Speciation and evolution of the *Capsicum* species

**Fig. 1:**
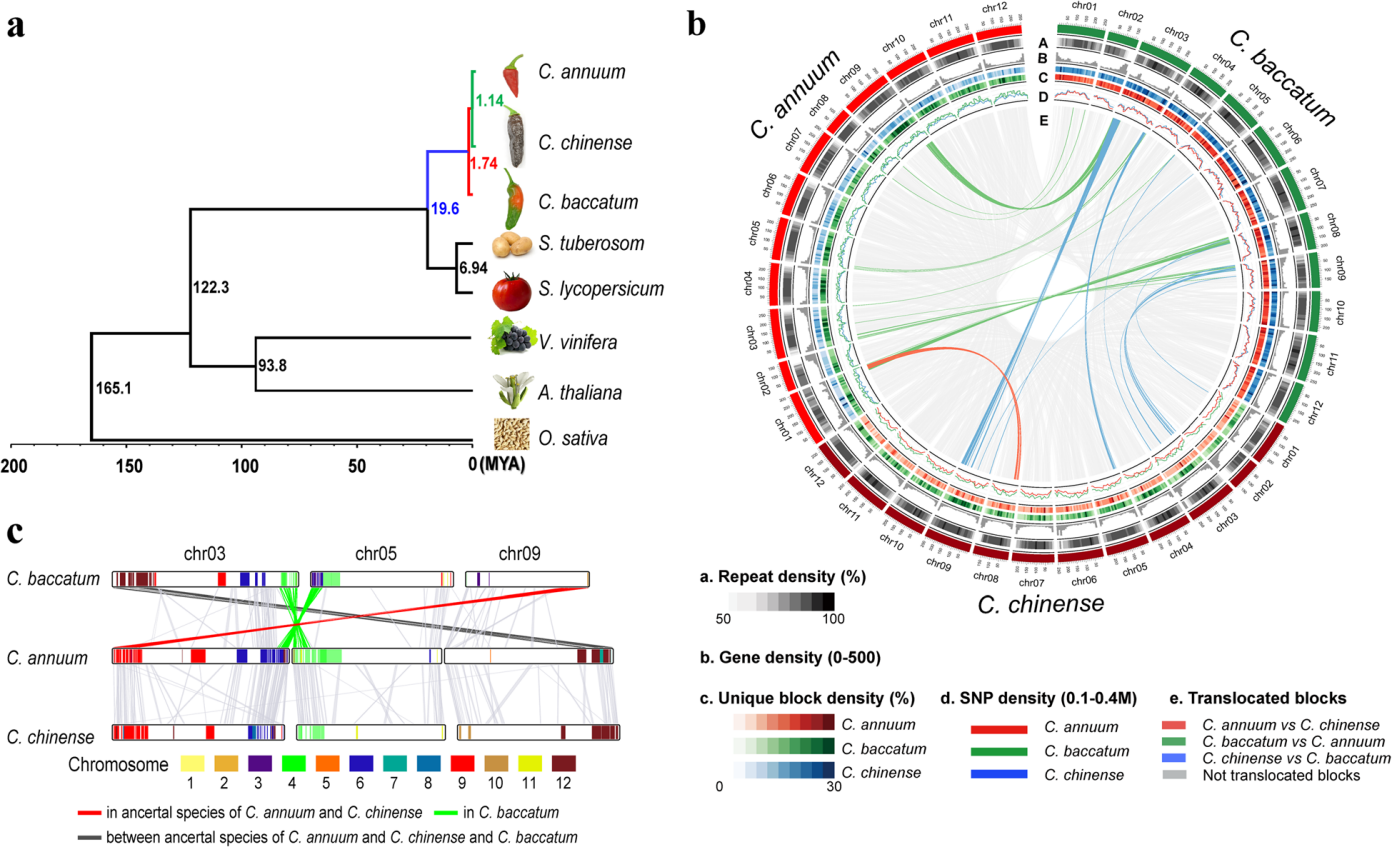
Lineage-divergence and genome structure comparisons of three *Capsicum* species. **a** The reconstructed *phylogenetic tree* of eight plant genomes indicates their evolutionary relationships and estimated divergence times. **b** The *circular diagram* shows the distribution of repeats, genes, genomic variations, and genome rearrangements in the pepper genomes. The subcategories indicate the density of repeats (A), genes (B), species-specific blocks (C), and SNPs (D) in the pepper genomes. The subcategory (E) depicts collinear and translocated blocks among the pepper genomes. **c** A linear comparison of the rearranged blocks in the pepper genomes. *Colors* in the *bars* indicate translocated regions when comparing to tomato and potato genomes. The *line colors* indicate translocations in the ancestral lineage leading to Annuum and Chinense (*red*), in Baccatum (*green*), and in the ancestor of Annuum and Chinense or Baccatum (*dark gray*)

**Fig. 2:**
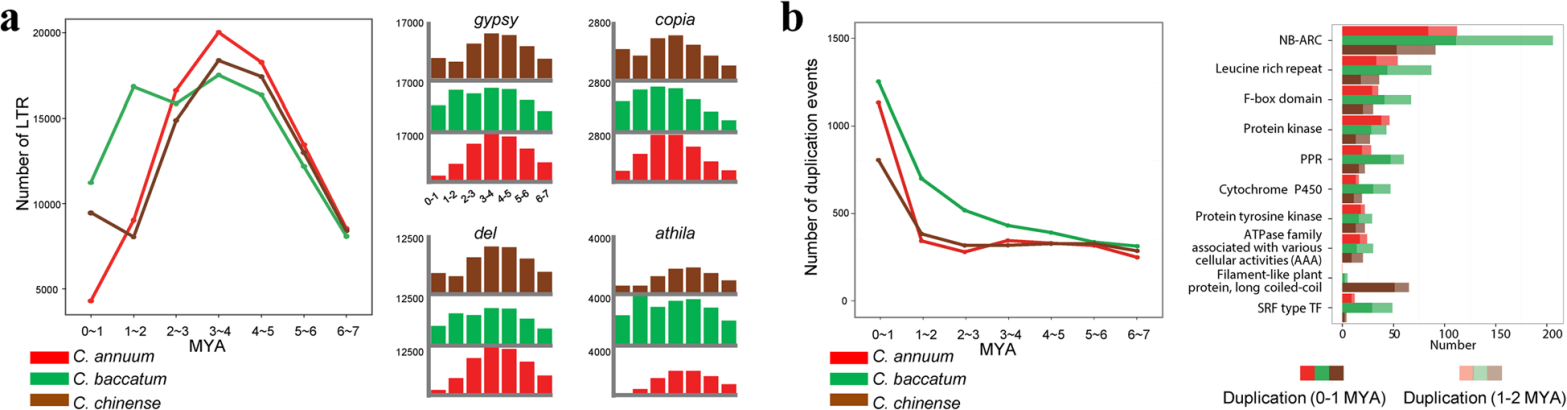
Evolutionary history of LTR-Rs and duplications of protein-coding genes in the pepper genomes. **a** Distribution of LTR-R insertions. The *graphs* in the *left* and *right panels* depict the predicted insertion dates of LTR-R superfamilies (*gypsy*, *copia*) and two specific families (*del*, *athila*). The *x-* and *y-*axes indicate the insertion times and the number of insertions at each time, respectively. **b** Time-scaled gene duplication history (*left*) and top ten repertoires of massive gene duplication (*right*). The *x-* and *y-*axes of the graph in the *left panel* indicate the approximate duplication time (MYA) and the number of gene duplications, respectively. The *x-* and *y-*axes of the histogram in the *right panel* represent the number of genes and domain description, respectively

A phylogenetic analysis of the peppers with other plant species revealed that the divergence among the three peppers occurred first between Baccatum and a progenitor of the other two peppers ~ 1.7 million years ago (MYA), followed by divergence between Annuum and Chinense lineages ~1.1 MYA (Fig. 1a; Additional file 1: Table S11). To identify genomic changes in the three pepper species, we compared the genome structures, LTR-R insertion patterns, and gene duplication histories across these pepper genomes (Fig. 1b, c and Fig. 2).

Chromosomal rearrangement is an important force in speciation, often producing unbalanced gametes that reduce hybrid fertility [28]. We performed an inter-genomic structural comparison and detected translocations among the pepper genomes (Fig. 1b). The results show that chromosomes 3, 5, and 9 exhibit translocations that differentiate Baccatum from the other two species (Fig. 1b, c). Collinearity comparisons among *Capsicum* species and two *Solanum* species revealed that the distal region on the long arm of chromosome 9 was conserved in Baccatum but translocated to the short arm of chromosome 3 in a shared ancestor of Annuum and Chinense (Fig. 1c; Additional file 1: Figure S5). Furthermore, chromosomes 6 and 4 of *Solanum* were detected in the terminal regions of the long and short arms of chromosomes 3 and 5 in Annuum and Chinense, respectively. In contrast, the orthologous regions of *Solanum* were mixed in the corresponding blocks of Baccatum (Fig. 1c). This indicates that the distal regions of the long and short arms of chromosomes 3 and 5 were translocated in the Baccatum lineage. We detected translocations between the terminal regions of the short arm of chromosome 3 in Baccatum and the long arm of chromosome 9 in Annuum and Chinense. Consequently, our analyses revealed that translocations have generated hetero karyotypes in both the Baccatum and the Annuum/Chinense progenitor lineages.

To compare LTR-R insertion patterns across the pepper genomes, we identified full-length LTR-Rs in each assembled genome and estimated their insertion times [29] (Additional file 1: Figure S5 and Table S12). A peak of LTR-R activity in Baccatum appeared around its speciation time 1–2 MYA (Fig. 2a). In particular, the *athila* family was highly amplified in Baccatum around the estimated speciation time, indicating that this subgroup may have explosively increased in Baccatum after speciation. In Chinense, we observed the recent proliferation of LTR-Rs (Fig. 2a).

Gene duplication is a major mechanism generating functional diversity between species by the creation of new genes [30, 31]. We detected recent gene duplication events and characterized the repertories of duplicated genes in the three pepper genomes during and after speciation (Fig. 2b). Overall, the duplication events were particularly frequent in the Baccatum lineage, both during and after the speciation. In particular, NLRs were extensively amplified in Baccatum in the last 0–2 MYA, and more recently in the other two peppers (Fig. 2b). Taken together, those results suggest that the chromosomal rearrangements, accumulation of specific LTR-Rs, and differential gene duplications have contributed to genome diversification in the *Capsicum*.

### Massive creation of new NLRs via LTR-R-driven retroduplication

**Fig. 3:**
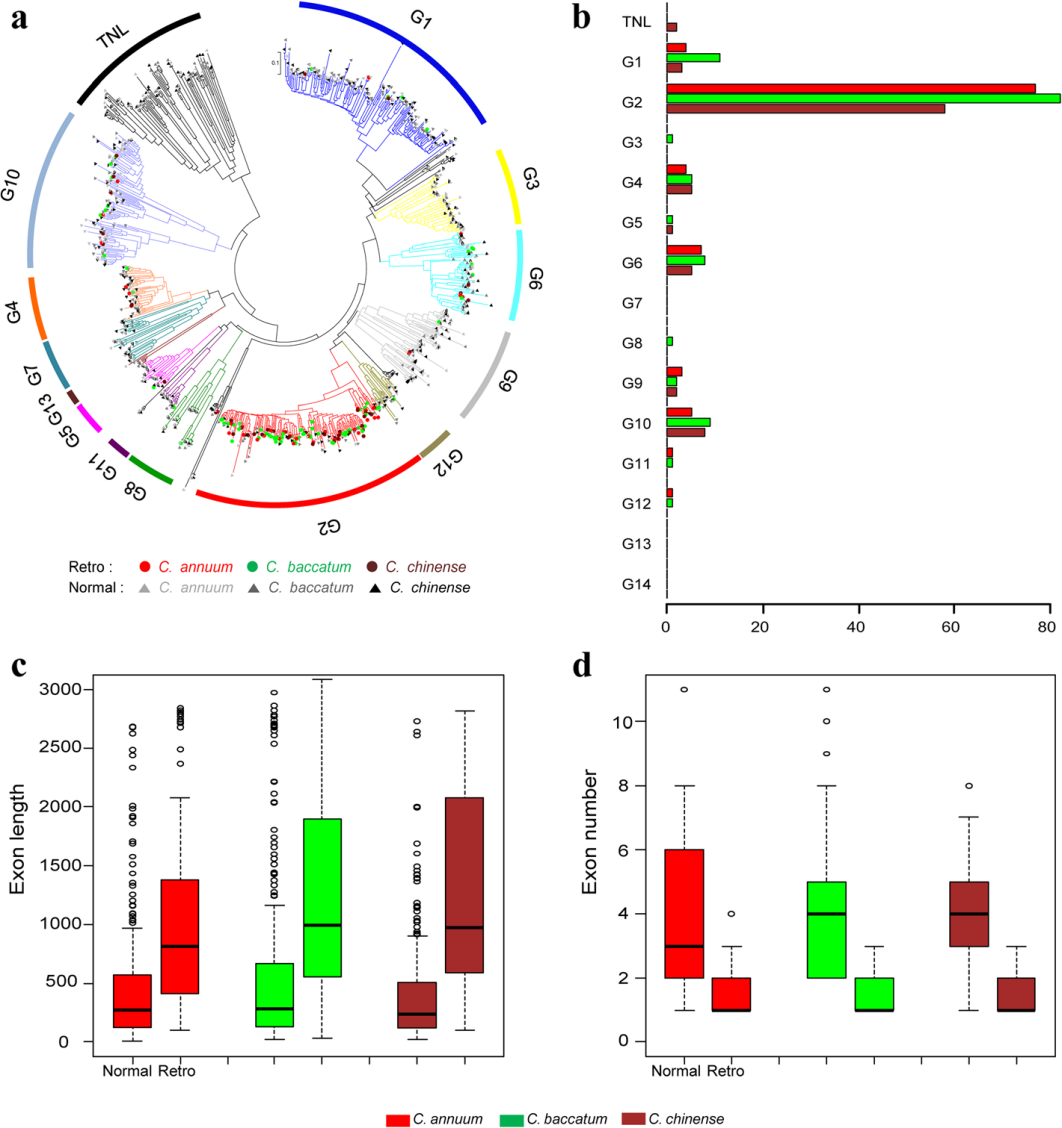
Emergence of large NLR gene families by retroduplication. **a** Intact NB-ARC domains of retroduplicated and normal NLRs are used for the *phylogenetic tree construction* as previously described method [21]. Each *color* of the phylogenetic tree indicates each subgroup. **b** The *bar graph* indicates the number of retroduplicated NLRs in each subgroup. The *x-* and *y-*axes indicate the numbers of genes and subgroups, respectively. **c**, **d** The exon lengths and the numbers of normal and retroduplicated NLRs are depicted. **c** The *x-* and *y-*axes indicate the normal and retroduplicated NLR groups and their exon lengths, respectively. **d** The *x-* and *y-*axes mean the groups of NLRs and the exon numbers, respectively

A previous study suggested that NLRs were amplified in pepper compared to tomato and potato genomes [21]. In particular, the coiled-coil NLR subgroup 2 (CNL-G2) was highly expanded in the pepper genome (Additional file 1: Table S13). To explore the possible mechanism of the NLR proliferation in *Capsicum* spp., we analyzed the NLRs and their flanking sequences. We identified 105, 123, and 86 NLRs located inside LTR-Rs in Annuum, Baccatum, and Chinense, respectively (Additional file 1: Figure S6, S7, and Table S13; Additional file 2: Table S14). Hence, a large proportion (~13%) of the NLRs were amplified by LTR-Rs, with the structures indicating that their retroduplicated origin is still intact. The retroduplicated NLRs were manly located on specific euchromatic chromosome arms (Additional file 1: Figure S8). Most of these NLRs (~70%) were in the CNL-G2 category, indicating that the copy number of specific NLR subfamilies was particularly expanded in specific chromosomes (Fig. 3a, b; Additional file 1: Figure S8). Furthermore, most of the retroduplicated NLRs (~ 72% of the total and ~ 67% of the CNL-G2 type) were inside non-autonomous LTR-Rs that contained no *gag* or *pol* protein coding potential (Additional file 1: Table S15). This suggests that all steps for the retroduplication, presumably including the initial sequence capture process, had to be provided *in trans*. To compare retroduplicated NLRs and NLRs which are not affected by LTR-Rs, we classified normal NLRs as false-negative annotations (Additional file 1: Table S13). We performed *Ka/Ks* analysis to compare selection pressure between retroduplicated and normal NLRs in CNL-G2. Our analysis revealed that both retroduplicated and normal NLRs were under purifying selection and *Ka/Ks* ratio of the both genes was not significantly different (Additional file 1: Figure S9).

When we compared the retroduplicated and normal NLRs in the CNL-G2 category, the number and length of exons were significantly fewer and longer in the retroduplicated NLRs, but not all of the retroduplicated NLRs have single exons (Fig. 3c, d). In total, ~ 32% of the retroduplicated NLRs in each species had multiple exons but all of those had a reduced number of introns compared to their predicted parental sequences (Additional file 1: Figure S7b, Table S15 and S16). For example, CB.v1.2.scaffold1410.1 having two exons is annotated as a retroduplicated NLR in CNG-G10 of Baccatum. We found that its potential parental sequence was consisted of six exons and sequence comparison of the both genes revealed that five exons of parental sequence were fully merged to the first exon (2.9 kbp) of CB.v1.2.scaffold1410.1 (Additional file 1: Figure S7b). These results suggest that retroduplicated NLRs containing introns in pepper genomes might be emerged through alternative splicing mechanisms such as intron retention or exon skipping as described in Zhang et al. [10].

We performed the genome-wide analyses using tomato, potato, and rice genomes to verify that the retroduplication is a general feature of genome evolution in the plant kingdom (Additional file 1: Table S13). We found that 21, 81, and 27 (8%, 18%, and 5%) of NLRs were inside LTR-Rs in tomato, potato, and rice, respectively (Additional file 1: Table S13). Of these, we identified parental sequences with multiple exons for 14, 71, and 16 of the NLRs inside LTR-Rs in tomato, potato, and rice, respectively, thus confirming their emergence by retroduplication (Additional file 1: Figure S10 and Table S17). Similar to the peppers, NLRs in a particular subgroup (CNL-G9) were primarily retroduplicated in potato (Additional file 1: Table S13). These results indicate that LTR-Rs played a key role in the expansion of NLRs by retroduplication throughout the plant kingdom and that the detected events are both recent and lineage-specific.

In addition to the NLRs, we looked for other genes inside LTR-Rs in the six plant species (Additional file 1: Table S18). In total, a range from 1398 genes in rice to 3898 genes in potato genomes were found to be inside LTR-Rs, suggesting possibility for emergence of a large proportion of genes in these plant species by LTR-R-driven duplication. On average, ~ 45% of them had functional domains including highly amplified families such as MADS-box TFs, cytochrome P450s, and protein kinases, and ~ 42% of those genes were expressed in one or more investigated tissues by RNA-sequencing (RNA-seq) analysis (Additional file 1: Table S18).

### Evolutionary mechanisms for the emergence of disease resistance genes in Solanaceae

The *L* genes encoded by the NLRs are known to provide resistance in peppers against *Tobamoviruses* and they belong to the CNL-G4 category, along with *I2* in tomato that provides resistance to race 2 of *Fusarium oxysporum* f. sp. *lycopersici* and *R3a* in potato that provides resistance to the late blight pathogen, *Phytophthora infestans* [32–34]. Each gene has single exon encoding a peptide of ~ 1300 amino acids. Synteny analysis and sequence comparison among pepper, potato, and tomato genomes suggested *L*, *I2*, and *R3a* are orthologous genes and the genomic regions containing *L*, *I2*, and *R3a* were tightly linked on chromosome 11 (Additional file 1: Figure S11a and Table S19). These results suggest the possibility that the genes originated by an early retroduplication and then underwent divergent evolution in each lineage.

**Fig. 4:**
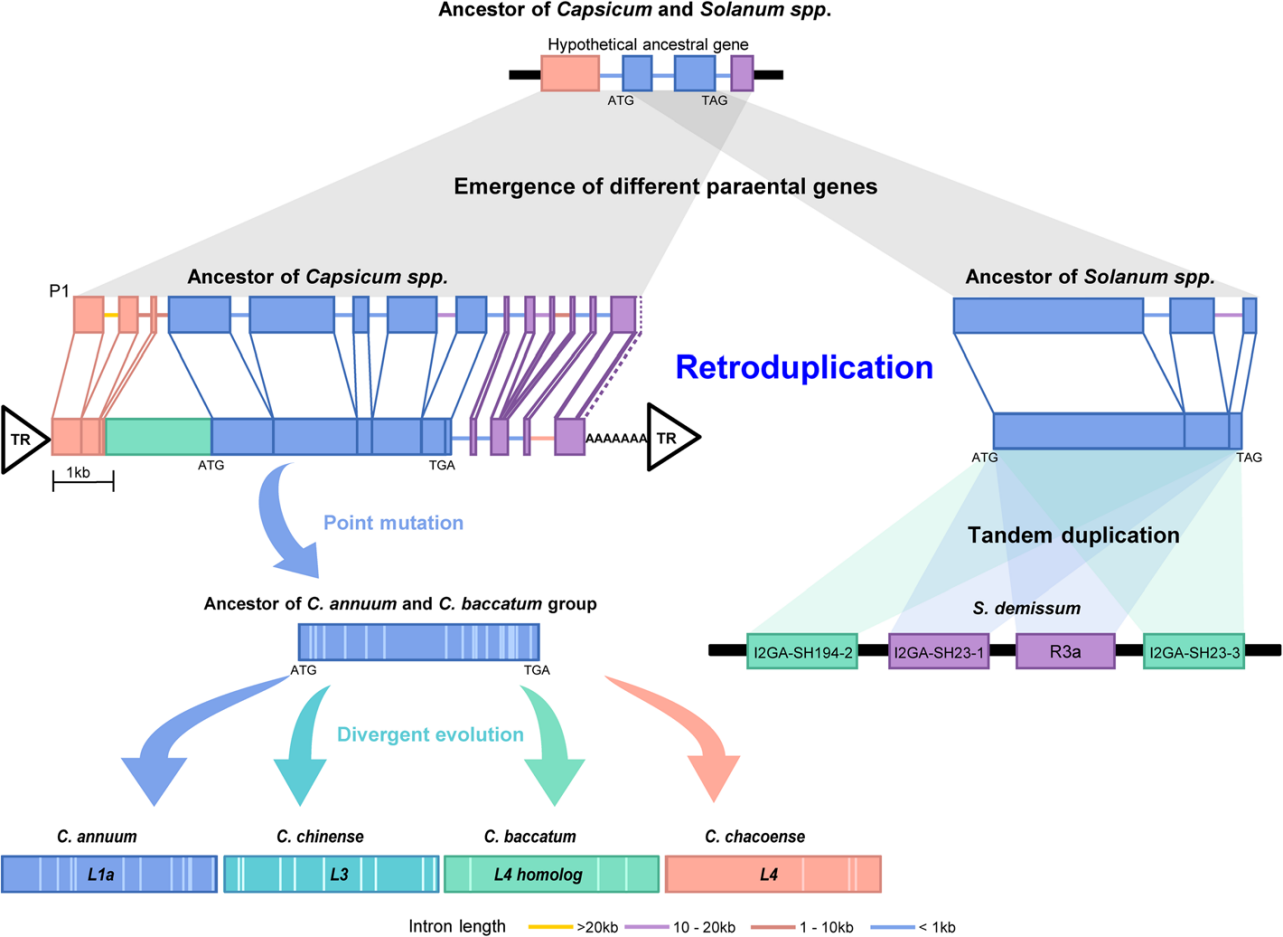
Emergence and evolution of *L* and *R3a* genes in the pepper and potato genomes. Models for the evolution of *L* and *R3a* in the pepper and potato are depicted. The gene names in the *R3a* cluster are from the previous analysis of Huang et al. [33]. The model proposes that *L* and *R3a* gene ancestors were first created by retroduplication, followed by the accumulation of point mutations and tandem duplication, respectively. DNA sequence indicative of a poly(A) tail and flanking terminal repeat (TR) sequences are depicted in the *diagram* as genomic evidence for a retroduplicated origin of *L*

We examined the evolutionary history of *L* genes and their putative parental genes in the pepper genomes (Fig. 4; Additional file 1: Figure S11b, c, and Table S20). The candidates for a parental gene (P1 to P6) were identified considering similarity, *Ks* values, and alignment coverage to *L* genes. All candidate parental sequences contained multiple exons. When candidate parental sequence P1 was compared with *L* in Annuum, the results suggested that *L* was derived from retroduplication in the ancestral lineage of *Capsicum* spp. ~ 8.9 MYA (Fig. 4). Because *L* has a 6.7-kb single exon, with only an intron in the 3′ UTR, and the presence of both flanking direct repeat sequences and a poly(A) “tail,” our analysis suggests that *L* emerged through capture and reverse transcription by a long interspersed nuclear element (LINE)-driven retroduplication (Fig. 4; Additional file 1: Figure S12). Sequence comparison of *L* genes in the three genomes and *L4* in *C. chacoense* revealed that the *L* genes were diversified by accumulation of lineage-specific sequence mutations after speciation within *Capsicum* (Fig. 4; Additional file 1: Table S21). Consequently, our results suggest that the ancestor of the *L* genes was derived from retroduplication and that subsequent divergent evolution has led to specific resistance against diverse strains of *Tobamovirus* in each species of *Capsicum* after speciation (Fig. 4).

To analyze the evolutionary processes acting on *R3a* of potato, we first performed a genome-wide search for the *R3a* as well as for candidate parental sequences. Because *R3a* is absent in the current potato reference genome [35], we could not carry out accurate comparisons of *R3a* and their homologs. However, *R3a* and its clustered genes originated from wild species, *Solanum demissum* [36], and were available in a public database. So, we compared these sequences with their closest homologs in the reference potato genome. Our analyses revealed that intronless sequences of the ancestral potato *R3a* might have emerged by RNA-based gene duplication in a shared ancestor of potato and tomato (Fig. 4). Subsequently, *R3a* and its paralogues were amplified by two rounds of tandem gene duplication after the divergence of potato and tomato (Fig. 4; Additional file 1: Table S22). Taken together, our results suggest that retroduplication events are a main evolutionary process in the emergence of new plant disease resistance genes, which can gain function via subsequent sequence variation and tandem duplication.

### Evolution of potential anthracnose resistance genes in Baccatum

**Fig. 5:**
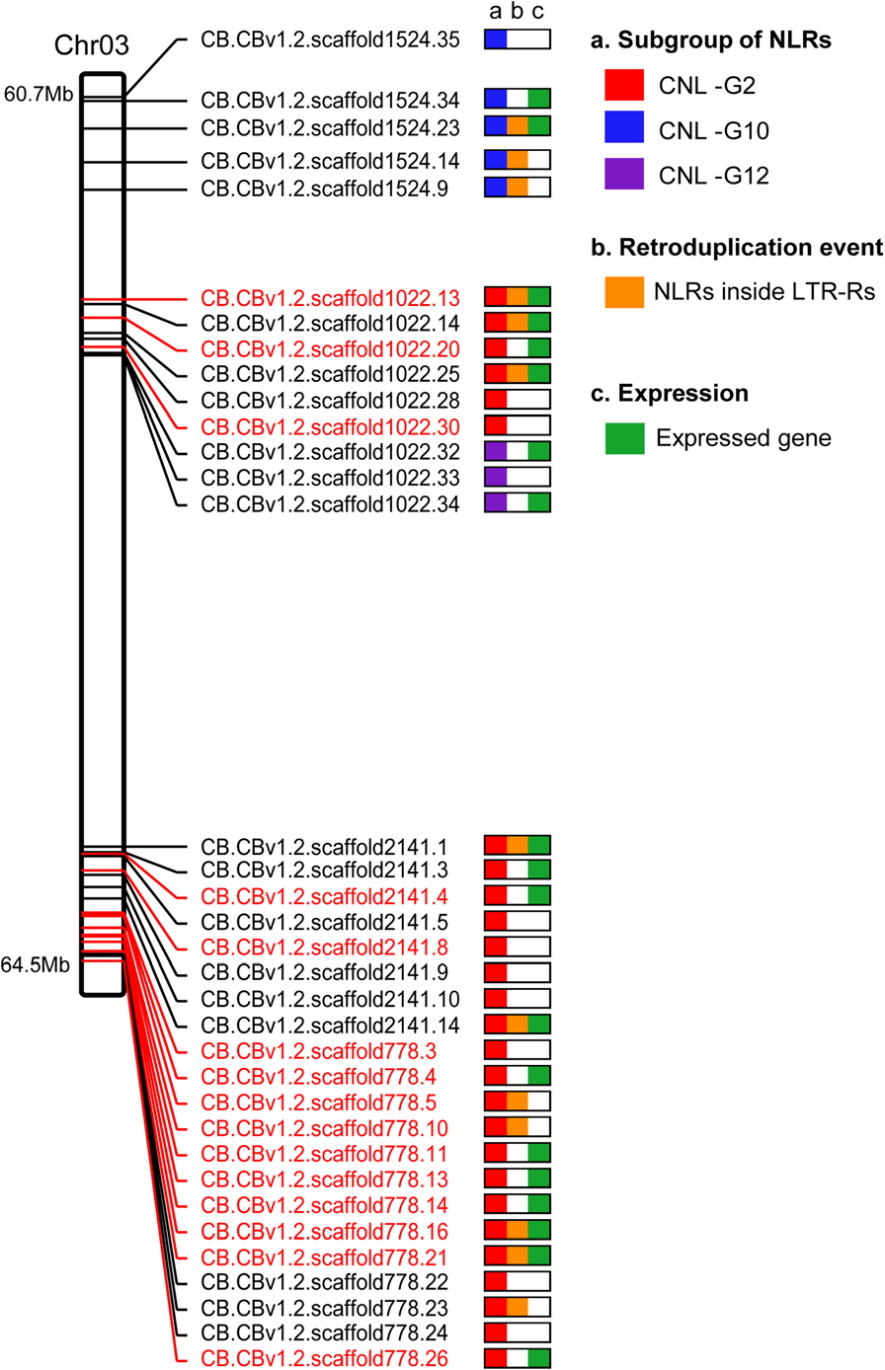
Potential anthracnose resistance genes in chromosome 3 of *C. baccatum*. Baccatum-specific NLRs in the major QTL region are visualized on 3.8 Mb of chromosome 3. The chromosome *plot* shows the subgroups, proposed retroduplication events, and expression results for the NLRs. The *black* and *red texts* indicate NLR IDs that emerged before and after the speciation of Baccatum, respectively

Pepper anthracnose caused by *Colletotrichum* spp. is one of the most devastating diseases in worldwide pepper production [37]. Due to the complexity of the interactions between the host and *Colletotrichum* spp. and the lack of resistance in the Annuum gene pool, a few Baccatum varieties were identified as the only breeding resources for anthracnose resistance [38]. Using pre-existing genetic information [39], we identified the pertinent genomic regions and obtained 64 NLRs from a 3.8 Mb region of Baccatum chromosome 3 as candidate resistance genes for *C. capsici* (Fig. 5; Additional file 1: Table S23). Previous studies reported that the main quantitative trait locus (QTL) for pepper resistance against *C. capsici* was located on chromosome 9 [39]; however, we found that QTL is located on chromosome 3 due to translocation in Baccatum and Annuum (Fig. 1c). We obtained 35 Baccatum-specific NLRs (27 in CNL-G2, five in CNL-G10, and three in CNL-G10) from the 64 NLRs by sequence comparison among the three pepper genomes (Fig. 5). Considering the gene duplication history, 15 of the 35 genes appear to have emerged after generation of the Baccatum lineage and all of them belong to the CNL-G2 category. Transcriptome evidence indicated that ten of those 15 genes are expressed in one or more tissues (Fig. 5; Additional file 1: Table S23). Furthermore, five of the 15 genes appear to have emerged by retroduplication (Fig. 5). Consequently, our results suggest that the retroduplication along with tandem and segmental duplications, may have played a major role in the emergence of the candidate genes for anthracnose resistance in the Baccatum lineage.

## Conclusions

In this study, we generated new and improved genome resources for three *Capsicum* species. Our data provide an accurate and updated gene model of the pre-existing reference pepper genome based on annotation with accumulated knowledge, highlighting the importance of genome improvement after the completion of sequencing project. High-quality chromosome-scale pseudomolecules constructed from three pepper genomes enabled precise comparisons of genome structures and evolutionary analyses, providing new insights into interspecies diversification via genome rearrangements and lineage-specific evolution of LTR-Rs and genes in the genus *Capsicum*. Furthermore, we found evidences of the evolution of NLRs by LTR-mediated retroduplication in dicot Solanaceae and monocot rice, suggesting that such phenomena are ubiquitous in angiosperms. Our results suggested that at least 5–18% of plant NLRs were emerged by LTR-R-driven retroduplication (Additional file 1: Table S13). A notable feature of this retroduplication is that distinct subfamilies of NLRs were highly retroduplicated in different plant lineages. We unveiled the emergence of functional disease resistance genes in the Solanaceae family by retroduplication and the subsequent neofunctionalization of those genes by dynamic evolutionary processes including lineage-specific sequence mutation and tandem duplication. Our data suggest that a large proportion of all genes (~4–10%) in plant species might have emerged by LTR-R-mediated duplication. We observed the lineage-specific amplification of specific gene families by LTR-Rs in various plant species, including such genes as those encoding cytochrome P450s in potato and MADS-box TFs in Baccatum (Additional file 1: Table S18). Taken together, our results provide new insights into the evolution of functional plant disease-resistance genes that belong to the NLR family as well as other high copy number gene families that are present in higher plants.

## Methods

### Genome assembly

In total, 425.7 Gb (132.2X coverage) and 526.7 Gb (136.1X coverage) of the Chinense and Baccatum genome sequences were generated in the Illumina Hiseq 2500 system (Additional file 1: Table S1). To remove unnecessary sequences for genome assembly, preprocessing analysis was performed as previously described [25] (Additional file 1: Table S2). The de novo genome assembly of each species was performed with SOAPdenovo2 [40] using the filtered raw sequences with parameters K = 77 and K = 81 for Chinense and Baccatum, respectively (Additional file 1: Table S3). The SSPACE software [41] was employed for additional scaffolding (-x 0 -m 46 -k 10 -a 0.4 -p 1); Gapcloser v.1.12 (GapCloser v112, http://soap.genomics.org.cn/down/GapCloser_release_2011.tar.gz) and Platanus [42] were implemented using default parameters to close gaps.

### Gene and repeat annotations

Gene annotation was performed for the three pepper genomes as described in Additional file 1 (Figure S2). To annotate protein-coding genes, we assembled transcripts using Tophat and Cufflinks [43] with the RNA-seq reads described in Additional file 1 (Table S7) and in a previous study [25]. The ISGAP pipeline [44] was used to extract accurate coding sequences from the assembled transcripts. Plant refSeq [45] and the public protein databases for *Arabidopsis* (TAIR 10), tomato (iTAG 2.3), potato (PGSC v3.4), and pepper (PGA v1.55) were used with Exonerate v2.2.0 [46] to align protein to the pepper genomes. Ab initio prediction was carried out with AUGUSTUS [47] version 3.0 using an in-house training set consisting of full-length complementary DNA generated from transcriptome analysis and by protein alignment. Consensus gene models were determined with EVM [48] and the biological description of each gene model was assigned based on the Uniprot database and INTERPRO scan v5.15-54.0 [49].

Repeat sequences were annotated in the initial contigs representing the estimated genome sizes and the assembled genomes of the three peppers, as shown in Additional file 1: Figure S4. An integrated repeat library of the three peppers was constructed using RepeatModeler (RepeatModeler, http://www.repeatmasker.org/RepeatModeler/). Annotation of intact LTR-Rs was performed using LTRHarvest [50] (-maxlenltr 2000 and –similar 80) and LTRDigest [51]. The subgroup of LTR-Rs in the integrated library was classified by comparing their sequences to those of the intact LTR-Rs using BLASTN (similarity > 90%) (Additional file 1: Figure S4).

### Comparison of genome structures

To identify regions that were either conserved or translocated between the *Capsicum* and *Solanum* species, we performed collinear analysis with MCScanX [52] using the gene models of the three peppers and the tomato and potato genomes described in Additional file 1 (Table S11). We identified regions that were not translocated between the tomato and potato genomes as conserved blocks in the *Solanum* species. The conserved blocks in the *Solanum* species were then compared to the three pepper genomes. Blocks in the pepper genomes that were conserved or translocated between the *Capsicum* and *Solanum* species were determined as shown in Additional file 1 (Figure S5). To investigate the translocated blocks in the three pepper genomes, we examined the gene collinearity for syntenic blocks as shown in Fig. 1c and Additional file 1 (Figure S5).

### Gene duplication history

To estimate the gene duplication times of the annotated genes in the pepper genomes, we constructed a computational pipeline by modifying a previously described method [53]. We first performed gene clustering analysis using OrthoMCL [54] to classify the gene family. We assumed that the genes in the same clusters were in the same family and performed all-by-all alignments of the coding sequences within the clusters in each species using PRANK [55]. For each alignment result, the *Ks* values were calculated using KaKs Calculator [56] and single-linkage clustering for the *Ks* values was performed using the hclust function in the R package. The molecular clock rate (*r*) was calculated to be 6.96 × 10^−9^ substitutions per synonymous site per year [57]. The duplication time was estimated using the formula, *Ks* value/2r.

### Estimation of divergence time

To estimate the divergence times of the plant genomes, we identified 2540 single copy genes in the rice, *Arabidopsis* (TAIR10), grape (VvGDB v2.0), tomato (v2.3), and potato (PGSC v3.4) genomes and the three pepper genomes using OrthoMCL clustering [54] (Additional file 1: Table S11). Multiple alignments of the single copy genes from the eight genomes were implemented using PRANK [55] (-f = nexus -codon). The speciation times of the eight plant species were calculated by phylogenetic analysis using the BEAST package [58].

### Evolutionary analyses of LTR-Rs

For the intact LTR-Rs, we performed alignment of the sequences between the 5′ and 3′ LTRs using PRANK. The DNA substitution rates (*K*) between the 5′ and 3′ LTRs were calculated using baseml in the PAML package [59]. The insertion times of the LTR-Rs were estimated using the formula, *K/2r* (*r* =1.3 × 10^−8^) considering higher substitution rate in intergenic regions than that in coding regions [60].

### Identification of retroduplicated NLRs in the plant genomes

To identify NLR genes inside LTR-Rs, we used the rice (MSU RGAP 7), potato (PGSC v3.4), and tomato (v2.3) genomes with the three pepper genomes. We first identified NLRs using a previously constructed pipeline [21] and extracted the NLRs within putative LTR-Rs predicted by LTRHarvest (Fig. 3a; Additional file 1: Table S13). We then compared those results with the repeats annotated by RepeatMasker and if the NLRs inside LTR-Rs overlapped with other TEs such as DNA transposons or Helitrons, we considered the LTR-Rs predicted by LTRHarvest to be incorrect and removed them. Because of rapid deletion of LTR-Rs and other unselected DNA in all flowering plants [14], we performed an additional identification of NLRs inside LTR-Rs using the annotated repeats including the partial LTR-Rs generated by RepeatMasker. We reasoned that if the NLRs were fully contained within LTR-Rs annotated by RepeatMasker, the NLRs were retroduplicated. To verify intron removal from the retroduplicated NLRs, we determined whether the candidate parental sequences of the NLRs contained multiple exons and had increased exon numbers by aligning the candidate parental sequences with the NLRs using Exonerate [46], requiring > 95% query coverage of the NLRs. To increase analysis accuracy, we excluded unclear cases where multi-exon NLRs having no parental sequences were detected inside LTR-Rs.To predict whole genes inside LTR-Rs in the six plant genomes, we performed genome-wide identification of possible structure of LTR-Rs using LTRHarvest, taking into account rapid sequence change between the LTRs (--similar 75%, minltrlen 100). Like annotation of the NLRs inside LTR-Rs, we extracted genes within directly repeated LTR regions as putatively retroduplicated genes. For the genes inside LTR-Rs, the number of expressed genes in one or more tissues was counted using RNA-seq data, as described in Additional file 1: Table S7 and in previous analyses [25, 35, 61, 62] (See Rice gene expression data, http://rice.plantbiology.msu.edu/ and Potato gene expression data, http://solanaceae.plantbiology.msu.edu/pgsc_download.shtml).

### Identification of false-negative and false-positive retroduplicated NLRs

We classified normal NLRs as false-negative annotations. From the total NLRs, we first excluded pre-identified retroNLRs. Of the remaining NLRs, the NLRs overlapped to LTR-regions (> 80% coverage) predicted by RepeatMasker were excluded. Finally, the remaining NLRs were considered as normal NLRs (Additional file 1: Table S13). To remove false-positive annotations among retroduplicated NLRs, we analyzed structure of the retroNLRs inside LTR-Rs in the six plant genomes. Because we detected cases which two or three genes were inside single LTR-Rs, we determined the criteria for accurate discrimination of retrogenes among the multiple genes inside single LTR-Rs (Additional file 1: Figure S6). We first excluded pre-annotated retroNLRs having no parental sequences then compared exon length and number of the remained genes. If the genes inside same LTR-R had the same exon number, we classified that a gene having the longest exon as a retrogene candidate (Additional file 1: Figure S6a). In case of genes having different exon numbers inside a single LTR-R, we selected the longest exon-containing gene (if the exon is > 1.5-fold larger than all of the exons in neighboring genes) as a retrogene candidate (Additional file 1: Figure S6b, c). If not, we selected the gene having the smallest exon number as a retrogene candidate (Additional file 1: Figure S6d). After manual confirmation, we filtered out 148 (25%) of 591 pre-identified retroNLR candidates in the six genomes. Finally, we determined 105, 123, 86, 21, 81, and 27 retroNLRs in Annuum, Baccatum, Chinense, tomato, potato, and rice genomes (Additional file 1: Figure S7, S10, and Table S13).

### Evolutionary investigation of functional disease-resistance genes in Solanaceae genomes

The *L*, *I2*, and *R3a* genes of pepper, tomato, and potato were used to investigate evolutionary processes acting on functional disease-resistance genes in the Solanaceae plants. The *L* genes in the *Capsicum* spp. were aligned to paralogues in the pepper genomes using Exonerate [46] and closest homologs were identified (Additional file 1: Table S20). All of the closest homologs in each species were found to contain multiple exons and a gene that we named P1 was identified as the most likely parental sequence. By comparison of the sequence divergence between P1 and its closest homologs in the other genomes, we confirmed that P1 was Annuum-specific (Additional file 1: Figure S11c). The 5′ and 3′ UTRs of *L1a* annotated based on RNA-seq evidence were also compared to the UTRs of P1 (Fig. 4).

For *R3a* in the potato, we aligned the coding sequences of *R3a* and genes within its cluster downloaded from GenBank (AY849382, AY849383, AY849384, and AY849385) to the potato genome. Because of the absence of those genes in the potato reference genome [35], we identified the closest homologs of *R3a* except *R3a* and its clustered genes in the potato genome. The duplication time of the *R3a* family was estimated by comparison of *R3a* and its homologs identified in the potato genome with the clustered genes. The *I2* sequence of tomato downloaded from GenBank was also used to search in tomato reference genome, but *I2* was not found.

### Identification of potential anthracnose resistance genes

To obtain candidate anthracnose resistance genes for *C. capsici*, we extracted NLRs located in the terminal region of the short arm of chromosome 3 of Baccatum based on pre-existing genetic information [39] (Additional file 1: Table S23). Candidate genes that may provide resistance in Baccatum against *C. capsici* were determined based on the degree of sequence conservation and the gene duplication time (Fig. 5).

## Additional files

Additional file 1: Figure S1–S12, Tables S1–S13 and S15–S23. (DOCX 1014 kb)

Additional file 2: Table S14. (XLSX 40 kb)

## References

[1] Bennetzen JL (2007). Patterns in grass genome evolution. Curr Opin Plant Biol.

[2] Kaessmann H, Vinckenbosch N, Long M (2009). RNA-based gene duplication: mechanistic and evolutionary insights. Nat Rev Genet.

[3] Wang W, Zheng H, Fan C, Li J, Shi J, Cai Z (2006). High rate of chimeric gene origination by retroposition in plant genomes. Plant Cell.

[4] Zhu Z, Tan S, Zhang Y, Zhang YE (2016). LINE-1-like retrotransposons contribute to RNA-based gene duplication in dicots. Sci Rep.

[5] Jiang N, Bao ZR, Zhang XY, Eddy SR, Wessler SR (2004). Pack-MULE transposable elements mediate gene evolution in plants. Nature.

[6] Morgante M, Brunner S, Pea G, Fengler K, Zuccolo A, Rafalski A (2005). Gene duplication and exon shuffling by helitron-like transposons generate intraspecies diversity in maize. Nat Genet.

[7] Ohshima K (2013). RNA-mediated gene duplication and retroposons: retrogenes, LINEs, SINEs, and sequence specificity. Int J Evol Biol.

[8] Lisch D (2013). How important are transposons for plant evolution?. Nat Rev Genet.

[9] Sakai H, Mizuno H, Kawahara Y, Wakimoto H, Ikawa H, Kawahigashi H (2011). Retrogenes in rice (*Oryza sativa* L. ssp *japonica*) exhibit correlated expression with their source genes. Genome Biol Evol.

[10] Zhang C, Gschwend AR, Ouyang Y, Long M (2014). Evolution of gene structural complexity: an alternative-splicing-based model accounts for intron-containing retrogenes. Plant Physiol.

[11] Zhu Z, Zhang Y, Long M (2009). Extensive structural renovation of retrogenes in the evolution of the *Populus* genome. Plant Physiol.

[12] Elrouby N, Bureau TE (2001). A novel hybrid open reading frame formed by multiple cellular gene transductions by a plant long terminal repeat retroelement. J Biol Chem.

[13] Jin YK, Bennetzen JL (1994). Integration and nonrandom mutation of a plasma-membrane proton atpase gene fragment within the *Bs1* retroelement of maize. Plant Cell.

[14] Ma J, Devos KM, Bennetzen JL (2004). Analyses of LTR-retrotransposon structures reveal recent and rapid genomic DNA loss in rice. Genome Res.

[15] Baucom RS, Estill JC, Chaparro C, Upshaw N, Jogi A, Deragon JM (2009). Exceptional diversity, non-random distribution, and rapid evolution of retroelements in the B73 maize genome. PLoS Genet.

[16] Jiang SY, Ramachandran S (2013). Genome-wide survey and comparative analysis of LTR retrotransposons and their captured genes in rice and sorghum. PLoS One.

[17] Hoen DR, Park KC, Elrouby N, Yu Z, Mohabir N, Cowan RK (2006). Transposon-mediated expansion and diversification of a family of *ULP*-like genes. Mol Biol Evol.

[18] Kong H, Landherr LL, Frohlich MW, Leebens-Mack J, Ma H, de Pamphilis CW (2007). Patterns of gene duplication in the plant *SKP1* gene family in angiosperms: evidence for multiple mechanisms of rapid gene birth. Plant J.

[19] Guo YL, Fitz J, Schneeberger K, Ossowski S, Cao J, Weigel D (2011). Genome-wide comparison of nucleotide-binding site-leucine-rich repeat-encoding genes in *Arabidopsis*. Plant Physiol.

[20] Ratnaparkhe MB, Wang XY, Li JP, Compton RO, Rainville LK, Lemke C (2011). Comparative analysis of peanut NBS-LRR gene clusters suggests evolutionary innovation among duplicated domains and erosion of gene microsynteny. New Phytol.

[21] Seo E, Kim S, Yeom SI, Choi D (2016). Genome-wide comparative analyses reveal the dynamic evolution of nucleotide-binding leucine-rich repeat gene family among Solanaceae Plants. Front Plant Sci.

[22] Hayashi K, Yoshida H (2009). Refunctionalization of the ancient rice blast disease resistance gene *Pit* by the recruitment of a retrotransposon as a promoter. Plant J.

[23] Kuykendall D, Shao J, Trimmer K (2009). A nest of LTR retrotransposons adjacent the disease resistance-priming gene *NPR1* in *Beta vulgaris* L. U.S. Hybrid H20. Int J Plant Genomics.

[24] Leister D (2004). Tandem and segmental gene duplication and recombination in the evolution of plant disease resistance gene. Trends Genet.

[25] Kim S, Park M, Yeom SI, Kim YM, Lee JM, Lee HA (2014). Genome sequence of the hot pepper provides insights into the evolution of pungency in *Capsicum* species. Nat Genet.

[26] Jeong HS, Jang S, Han K, Kwon JK, Kang BC (2015). Marker-assisted backcross breeding for development of pepper varieties (*Capsicum annuum*) containing capsinoids. Mol Breed.

[27] Lee YR, Yoon JB, Lee J (2016). A SNP-based genetic linkage map of *Capsicum baccatum* and its comparison to the *Capsicum annuum* reference physical map. Mol Breed.

[28] Rieseberg LH (2001). Chromosomal rearrangements and speciation. Trends Ecol Evol.

[29] SanMiguel P, Gaut BS, Tikhonov A, Nakajima Y, Bennetzen JL (1998). The paleontology of intergene retrotransposons of maize. Nat Genet.

[30] Flagel LE, Wendel JF (2009). Gene duplication and evolutionary novelty in plants. New Phytol.

[31] Panchy N, Lehti-Shiu M, Shiu SH (2016). Evolution of gene duplication in plants. Plant Physiol.

[32] Simons G, Groenendijk J, Wijbrandi J, Reijans M, Groenen J, Diergaarde P (1998). Dissection of the fusarium *I2* gene cluster in tomato reveals six homologs and one active gene copy. Plant Cell.

[33] Huang S, van der Vossen EA, Kuang H, Vleeshouwers VGAA, Zhang N, Borm TJ (2005). Comparative genomics enabled the isolation of the *R3a* late blight resistance gene in potato. Plant J.

[34] Tomita R, Sekine KT, Mizumoto H, Sakamoto M, Murai J, Kiba A (2011). Genetic basis for the hierarchical interaction between *Tobamovirus* spp. and *L* resistance gene alleles from different pepper species. Mol Plant Microbe Interact.

[35] Potato Genome Sequencing Consortium (2011). Genome sequence and analysis of the tuber crop potato. Nature.

[36] Huang S, Vleeshouwers VGAA, Werij JS, Hutten RCB, van Eck HJ, Visser RGF (2004). The *R3* resistance to *Phytophthora infestans* in potato is conferred by two closely linked R genes with distinct specificities. Mol Plant Microbe Interact.

[37] Than PP, Prihastuti H, Phoulivong S, Taylor PW, Hyde KD (2008). Chilli anthracnose disease caused by *Colletotrichum* species. J Zhejiang Univ Sci B.

[38] Mahasuk P, Struss D, Mongkolporn O (2016). QTLs for resistance to anthracnose identified in two *Capsicum* sources. Mol Breed.

[39] Lee J, Do JW, Yoon JB (2011). Development of STS markers linked to the major QTLs for resistance to the pepper anthracnose caused by *Colletotrichum acutatum* and *C. capsici*. Hortic Environ Biotechnol.

[40] Luo R, Liu B, Xie Y, Li Z, Huang W, Yuan J (2012). SOAPdenovo2: an empirically improved memory-efficient short-read *de novo* assembler. Gigascience.

[41] Boetzer M, Henkel CV, Jansen HJ, Butler D, Pirovano W (2011). Scaffolding pre-assembled contigs using SSPACE. Bioinformatics.

[42] Kajitani R, Toshimoto K, Noguchi H, Toyoda A, Ogura Y, Okuno M (2014). Efficient *de novo* assembly of highly heterozygous genomes from whole-genome shotgun short reads. Genome Res.

[43] Ghosh S, Chan CK (2016). Analysis of RNA-Seq data using TopHat and Cufflinks. Methods Mol Biol.

[44] Kim S, Kim MS, Kim YM, Yeom SI, Cheong K, Kim KT (2015). Integrative structural annotation of *de novo* RNA-Seq provides an accurate reference gene set of the enormous genome of the onion (*Allium cepa* L.). DNA Res.

[45] Pruitt KD, Tatusova T, Brown GR, Maglott DR (2012). NCBI Reference Sequences (RefSeq): current status, new features and genome annotation policy. Nucleic Acids Res.

[46] Slater GS, Birney E (2005). Automated generation of heuristics for biological sequence comparison. BMC Bioinformatics.

[47] Stanke M, Tzvetkova A, Morgenstern B (2006). AUGUSTUS at EGASP: using EST, protein and genomic alignments for improved gene prediction in the human genome. Genome Biol.

[48] Haas BJ, Salzberg SL, Zhu W, Pertea M, Allen JE, Orvis J (2008). Automated eukaryotic gene structure annotation using EVidenceModeler and the program to assemble spliced alignments. Genome Biol.

[49] McDowall J, Hunter S (2011). InterPro protein classification. Methods Mol Biol.

[50] Ellinghaus D, Kurtz S, Willhoeft U (2008). LTRharvest, an efficient and flexible software for *de novo* detection of LTR retrotransposons. BMC Bioinformatics.

[51] Steinbiss S, Willhoeft U, Gremme G, Kurtz S (2009). Fine-grained annotation and classification of *de novo* predicted LTR retrotransposons. Nucleic Acids Res.

[52] Wang Y, Tang H, Debarry JD, Tan X, Li J, Wang X (2012). MCScanX: a toolkit for detection and evolutionary analysis of gene synteny and collinearity. Nucleic Acids Res.

[53] Kim SB, Kang WH, Huy HN, Yeom SI, An JT, Kim S (2017). Divergent evolution of multiple virus-resistance genes from a progenitor in *Capsicum* spp. New Phytol.

[54] Li L, Stoeckert CJ, Roos DS (2003). OrthoMCL: identification of ortholog groups for eukaryotic genomes. Genome Res.

[55] Loytynoja A (2014). Phylogeny-aware alignment with PRANK. Methods Mol Biol.

[56] Zhang Z, Li J, Zhao XQ, Wang J, Wong GK, Yu J (2006). KaKs_Calculator: calculating Ka and Ks through model selection and model averaging. Genomics Proteomics Bioinformatics.

[57] de Sa Moniz M, Drouin G (1996). Phylogeny and substitution rates of angiosperm actin genes. Mol Biol Evol.

[58] Drummond AJ, Suchard MA, Xie D, Rambaut A (2012). Bayesian phylogenetics with BEAUti and the BEAST 1.7. Mol Biol Evol.

[59] Yang Z (2007). PAML 4: phylogenetic analysis by maximum likelihood. Mol Biol Evol.

[60] Ma J, Bennetzen JL (2004). Rapid recent growth and divergence of rice nuclear genomes. Proc Natl Acad Sci.

[61] The Tomato Genome Consortium (2012). The tomato genome sequence provides insights into fleshy fruit evolution. Nature.

[62] Goff SA, Ricke D, Lan TH, Presting G, Wang R, Dunn M (2002). A draft sequence of the rice genome (*Oryza sativa* L. ssp. *japonica*). Science.

[63] Kim S, Park J, Yeom SI, Kim YM, Seo E, Kim KT, et al. New reference genome sequences of hot pepper reveal the massive evolution of plant disease-resistance genes by retroduplication. GenBank. https://www.ncbi.nlm.nih.gov/nuccore/AYRZ00000000. 10.1186/s13059-017-1341-9 PMC5664825 29089032

[64] Kim S, Park J, Yeom SI, Kim YM, Seo E, Kim KT, et al. New reference genome sequences of hot pepper reveal the massive evolution of plant disease-resistance genes by retroduplication. GenBank. https://www.ncbi.nlm.nih.gov/nuccore/MLFT00000000. 10.1186/s13059-017-1341-9 PMC5664825 29089032

[65] Kim S, Park J, Yeom SI, Kim YM, Seo E, Kim KT, et al. New reference genome sequences of hot pepper reveal the massive evolution of plant disease-resistance genes by retroduplication. GenBank. https://www.ncbi.nlm.nih.gov/nuccore/MCIT00000000. 10.1186/s13059-017-1341-9 PMC5664825 29089032

