# Supplementary material for: New reference genome sequences of hot pepper reveal the massive evolution of plant disease-resistance genes by retroduplication
Source: Genome Biol. 2017 Nov 1;18:210. doi: 10.1186/s13059-017-1341-9 (PMC5664825; doi:10.1186/s13059-017-1341-9)
Supplement: Supplementary file 1 — (DOCX 1014 kb) [file 13059_2017_1341_MOESM1_ESM.docx]

**
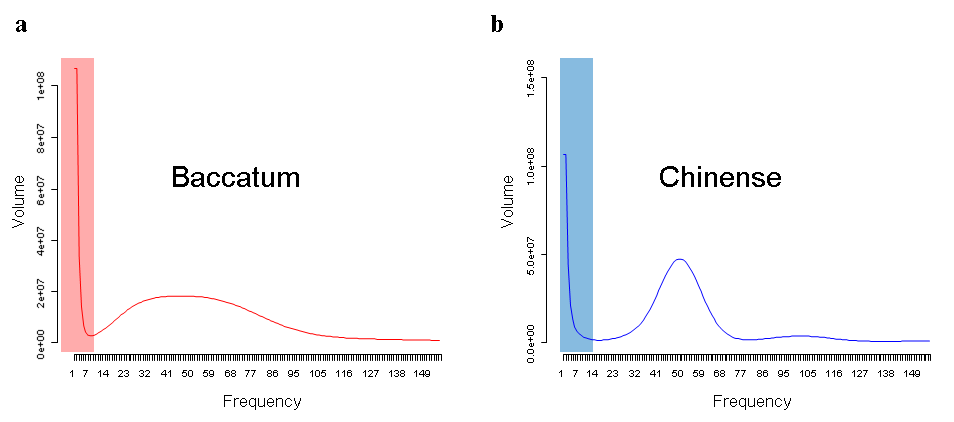
**

**Figure S1. The 19-mer distribution patterns for the Baccatum and Chinense genome sequences.** **(a-b)** The *x-* and *y-*axes indicate the frequency and volume of 19-mers, respectively. Shaded regions in the graphs indicate low-frequency data as erroneous candidates.

**
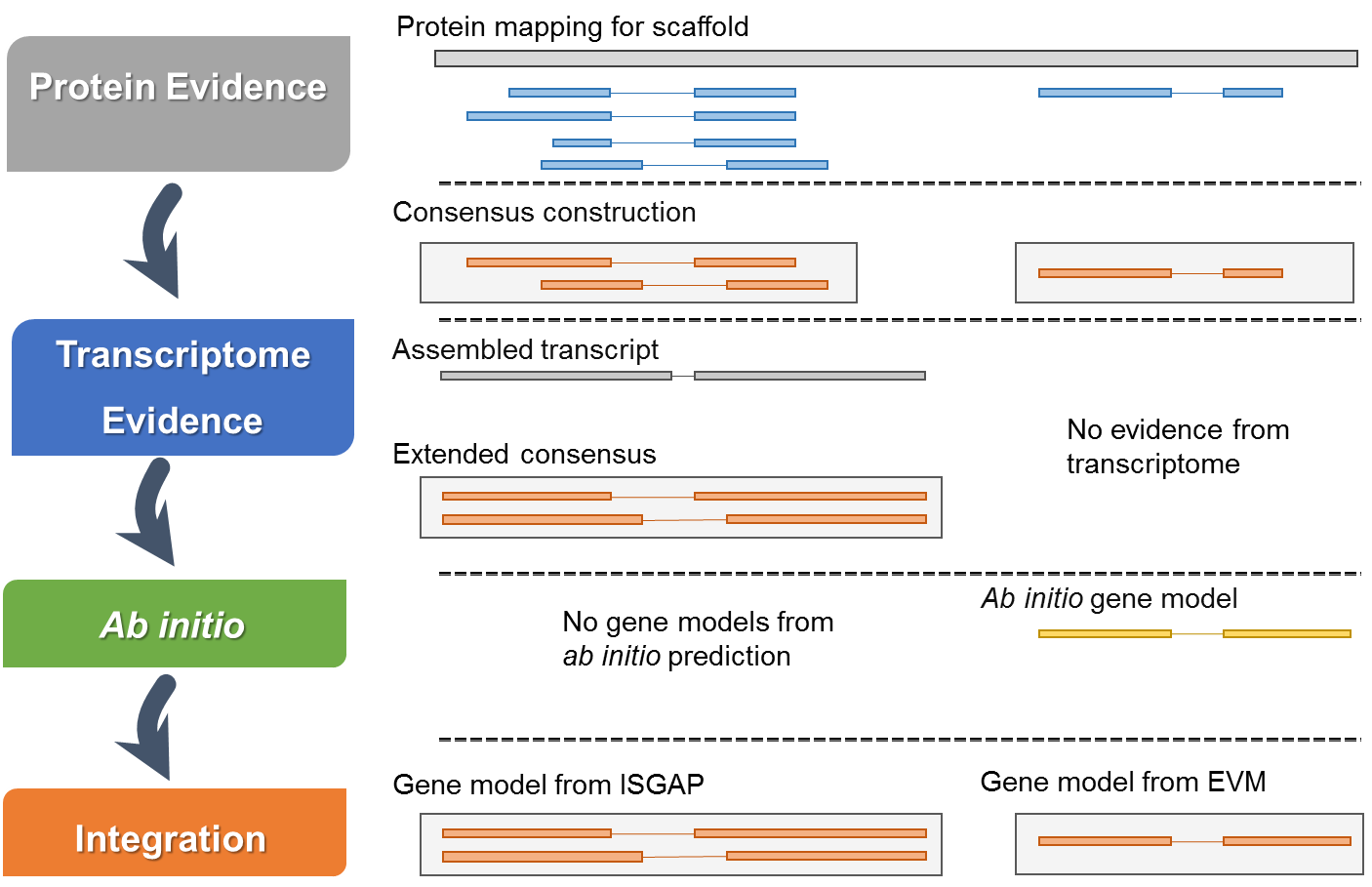
**

**Figure S2. Gene annotation scheme for the pepper genomes.** The diagram depicts our gene annotation process for the pepper genomes using proteins, transcriptome and *ab initio* prediction. ISGAP[[1](#_ENREF_1)] identifies gene structure based on transcriptome and protein evidences. EVM[[2](#_ENREF_2)] integrates results of protein mapping and / or *ab initio* prediction using AUGUSTUS[[3](#_ENREF_3)].**
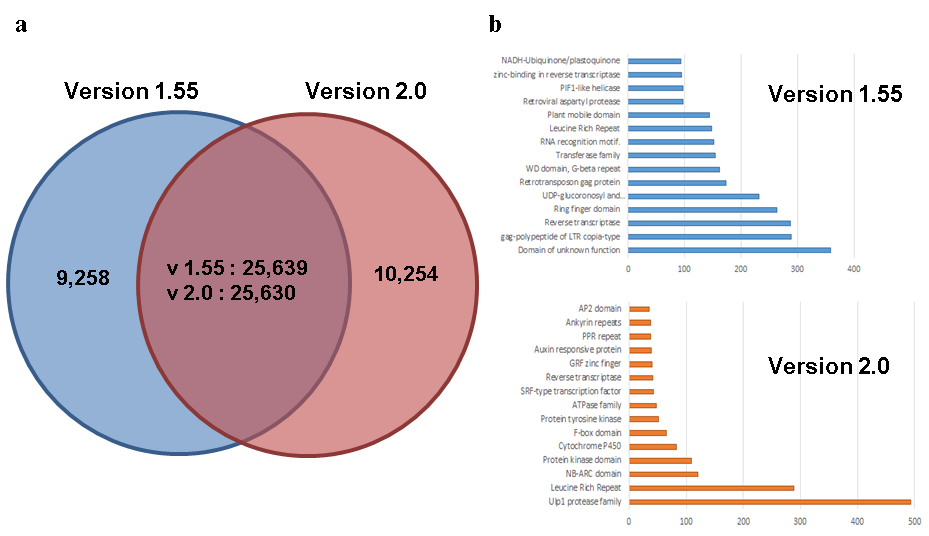
**

**Figure S3. Comparison of annotated gene sets between the pre-existing and newly generated versions of the Annuum genome.** **(a)** The Venn diagram indicates the numbers of overlapping and non-overlapping gene models between versions 1.55[[4](#_ENREF_4)] and 2.0, considering the genomic positions. **(b)** The bar graphs show the numbers of predicted genes in the top 15 domain descriptions in non-overlapping gene models in v1.55 and v2.0. The *x-* and *y-*axes indicate the number of annotated genes with a particular domain and the category of domain, respectively.

**
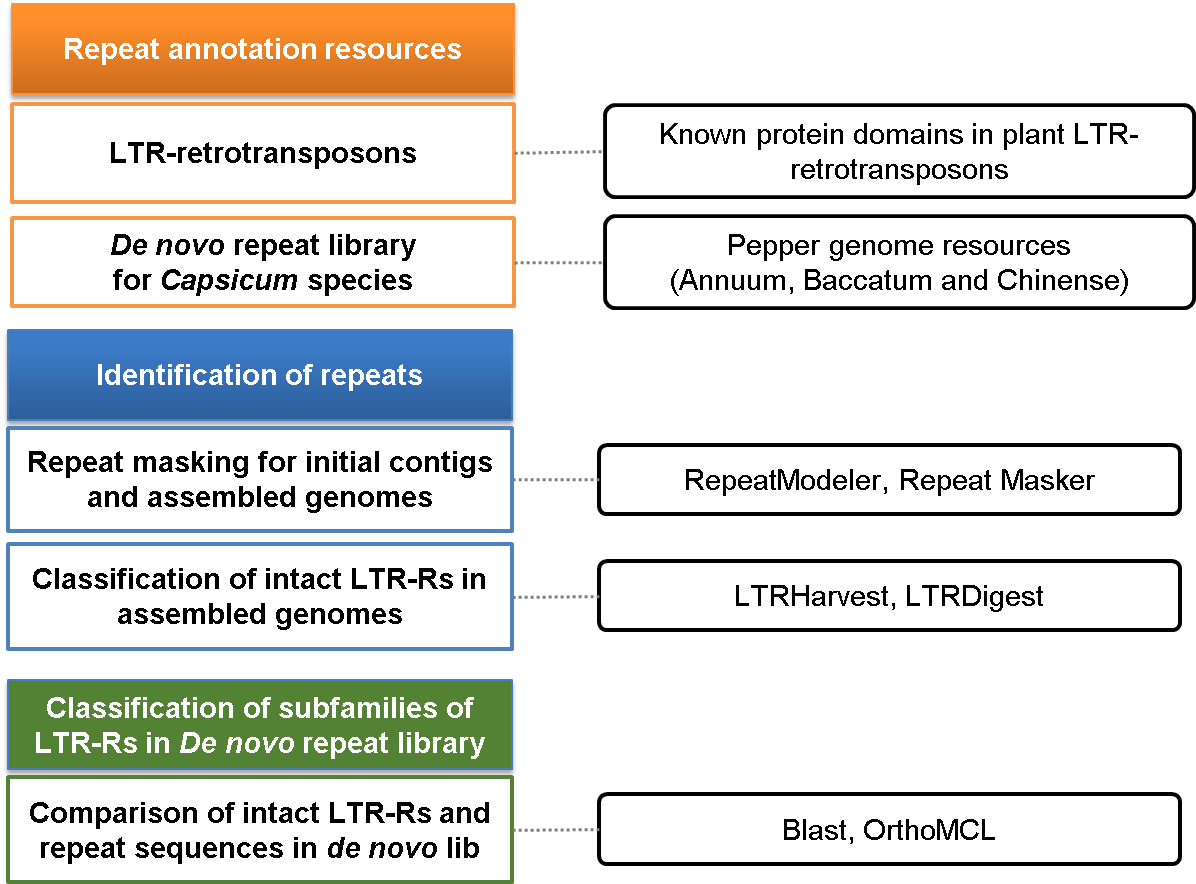
**

**Figure S4. The pipeline used for annotation of repeats in the pepper genomes.** The diagram depicts three steps in repeat annotation, i) construction of repeat libraries using repeat annotation resources, ii) identification of repeats and intact LTR-Rs in the pepper genomes, and iii) assignment of family information for LTR-Rs in the *de novo* repeat library. The diagrams in the right panel indicate the resources and tools used for the annotation of repeats.

**
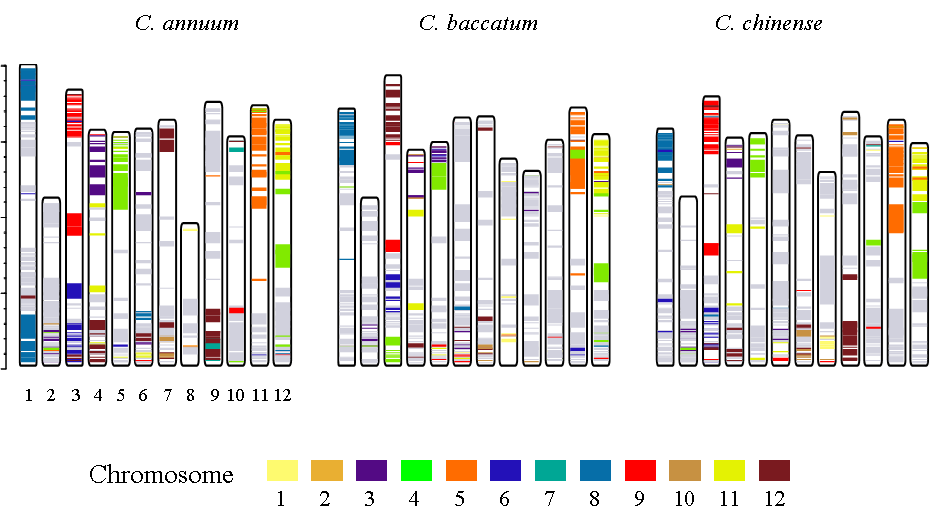
**

**Figure S5. The collinear blocks in pepper genomes exhibiting conserved syntenic regions between tomato and potato genomes.** The colours in the bars indicate collinear blocks in the pepper genomes that are also conserved as blocks within the tomato and potato genomes. Chromatic and grey colours indicate translocated and non-translocated regions, respectively, between the *Capsicum* and *Solanum* genomes.

**
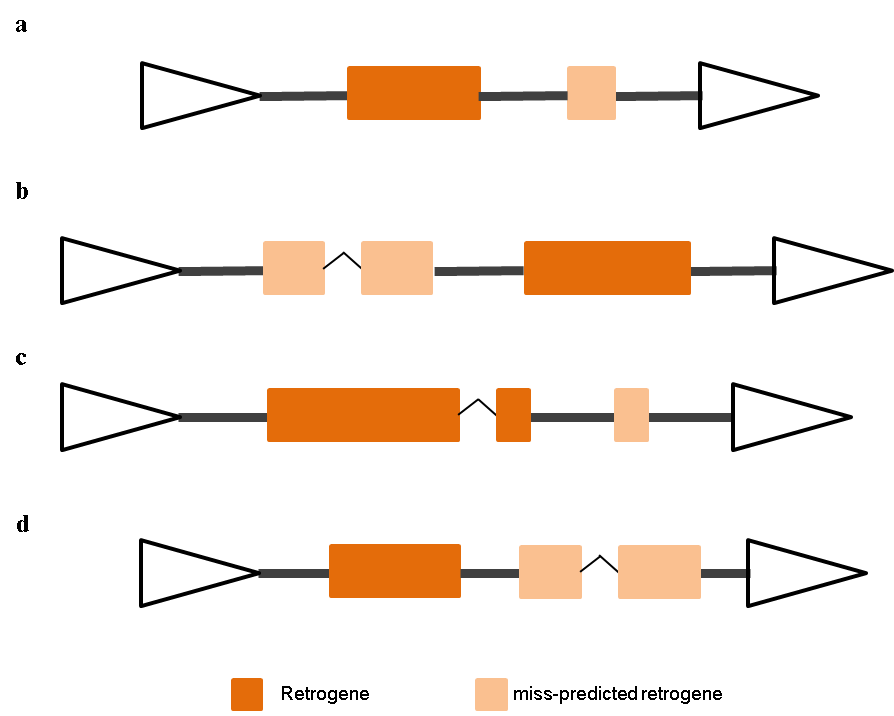
**

**Figure S6. Discrimination for erroneous annotations of retrogenes inside LTR-Rs.** Possible cases for multiple genes inside single LTR-R are depicted as diagrams. Triangles and rectangles mean LTRs and genes, respectively. Bold black bar indicates intergenic region and diagonal line means intron sequences **(a)** A case of multiple genes having same exon number inside single LTR-R **(b-d)** Cases of multi-genes having different exon number inside single LTR-R.

**
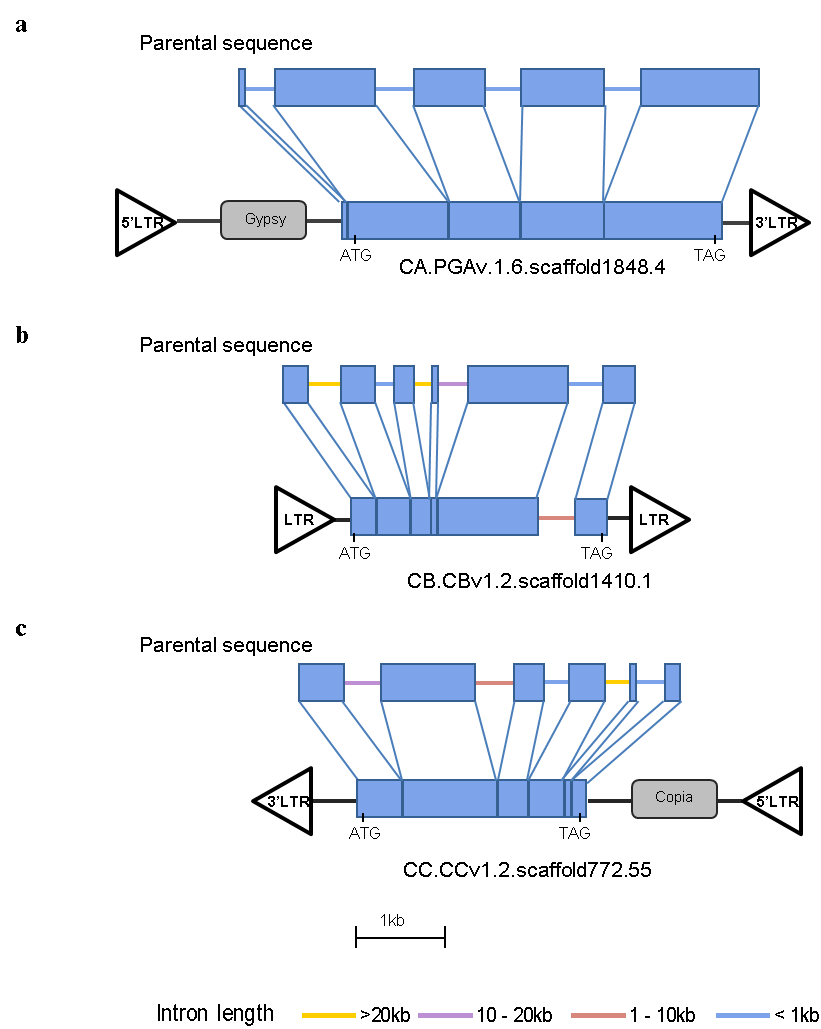
**

**Figure S7. Emergence of retroNLRs in *Capsicum* spp.** The diagrams indicate models for emergence of retroNLRs containing single or multiple exons inside LTR-Rs in pepper genomes. Transcript sequences are compared to their parental sequences. Position of 5’ and 3’ LTRs are determined considering direction of the retrotransposon proteins. When the direction of LTR-R is uncertain, LTRs are depicted without marks for 5’ and 3’ **(a)** Comparison of retroNLR having single exon inside Ty3-*gypsy* element and its parental sequence containing five exons in Annuum. **(b)** A case of retroduplication for multi-exon NLR inside potential non-autonomous family in Baccatum. **(c)** An example of retroduplication event by Ty1-*copia* element in Chinense.

**
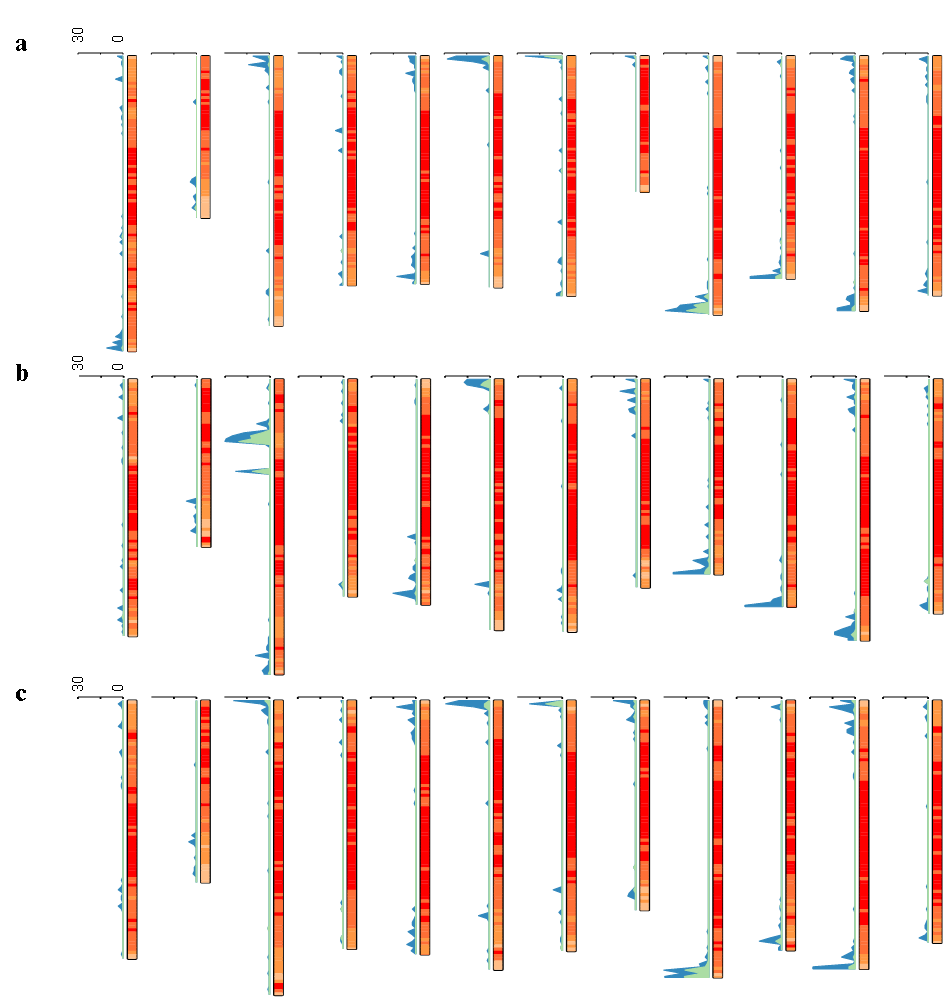
**

**
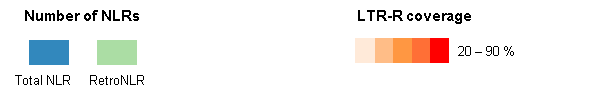
**

**Figure S8. Chromosomal localization of LTR-Rs and NLR genes in *Capsicum* spp.** Chromosome-wide distribution of LTR-Rs and NLRs is depicted as diagrams. The histogram and heatmap show the number of retroduplicated and total NLRs and density of LTR-Rs, respectively.

**
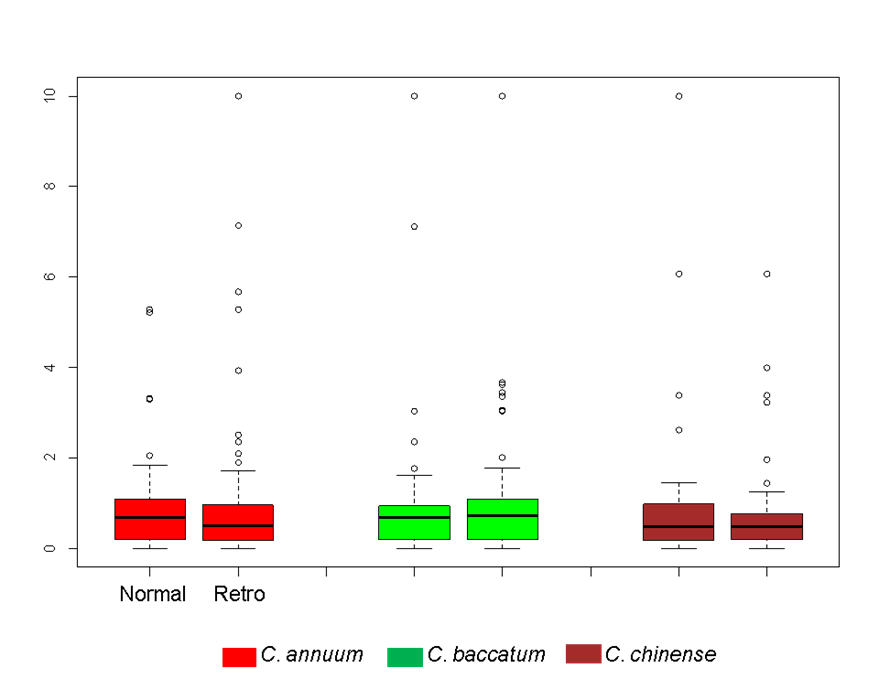
**

**Figure S9. Comparison of *Ka/Ks* ratio for retroduplicated and normal NLRs in CNL-G2.** The box-plot indicates distribution of the *Ka/Ks* ratio for retroduplicated and normal NLRs. The x- and y-axis indicate type of NLR sand *Ka/Ks* ratio, respectively.

**
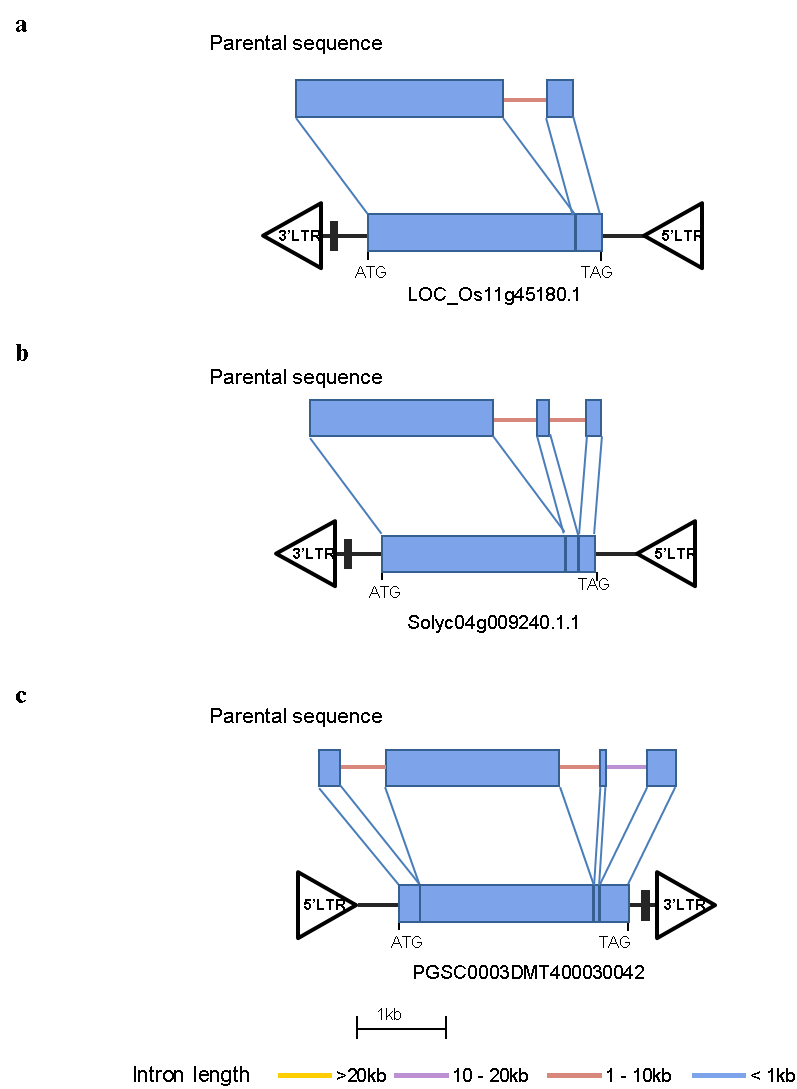
**

**Figure S10. LTR-R mediated retroduplications of NLRs in rice, tomato and potato genomes.** The diagram depicts models of retroduplication for NLRs inside LTR-Rs in rice, tomato and potato genomes. The black box in front of 3’LTR indicates PPT regions and the direction of LTR-Rs is determined based on the PPT position.

**
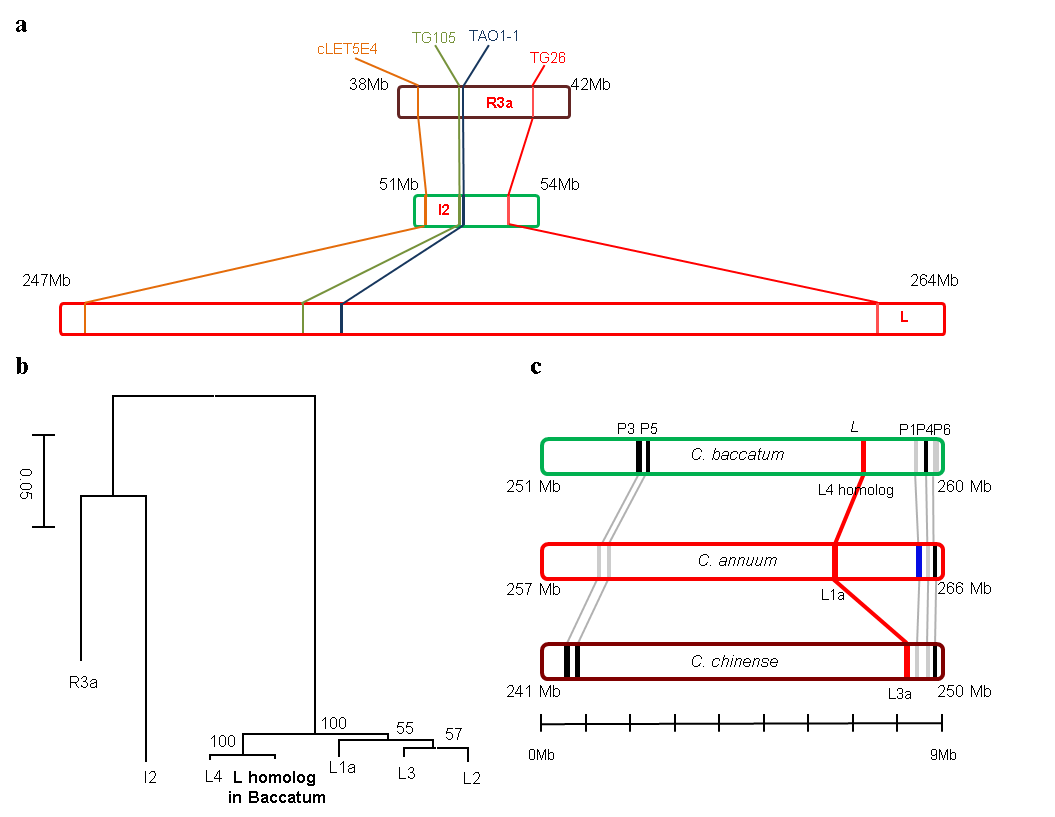
**

**Figure S11. Comparative analyses of *R3a*, *I2* and *L* genes and the locations of those genes in the Solanaceae genomes.** **(a)** Syntenic regions including *L*, *I2*, and *R3a* in the pepper, tomato and potato genomes are depicted as bar graphs. The marker names were described in a previous study[[5](#_ENREF_5)]. **(b)** The phylogenetic tree of *L*, *I2* and *R3a*. **(c)** Bar graphs depict the locations of the closet homologs (P1 to P6) of the *L* genes in the pepper genomes as candidate parental sequences. Black and grey lines in the bars mean the presence and absence of the parental sequences in pepper genomes, respectively. The blue band within the red bar indicates the location of P1 in Annuum, the most likely parental gene of *L1a*.

**
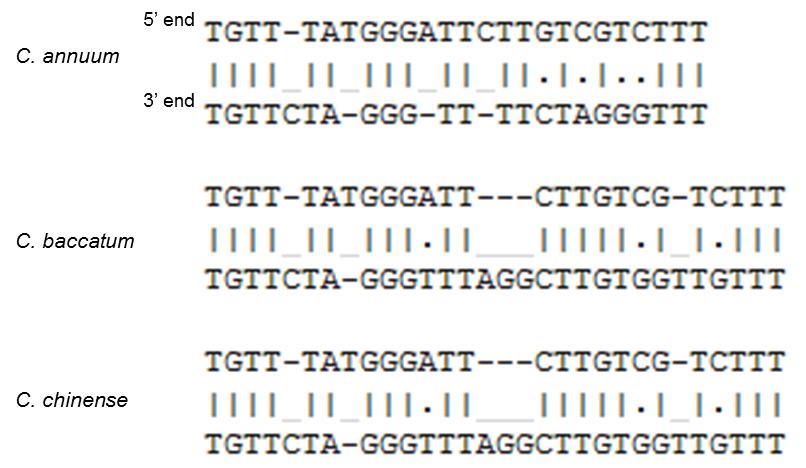
**

**Figure S12. Alignments between the 5’ and 3’ end direct repeat sequences flanking the *L* genes in the pepper genomes.**

**Table S1.** Generated pepper genome sequences in this study.

| Sequencing  data | Insert  size | *C. baccatum* PBC81 | | | *C. chinense* PI159236 | |
| --- | --- | --- | --- | --- | --- | --- |
|  |  | Length  (Depth) | Read  length (bp) | Length  (Depth) | | Read  length (bp) |
| Illumina  reads | 200 bp | 221.1 (57.1) | 151 | 98.9 (30.7) | | 101 |
|  | 400 bp | 73.6 (19.0) | 151 | 94.7 (29.4) | | 101 |
|  | 600 bp | 72.1 (18.6) | 151 | 96.1 (29.8) | | 101 |
|  | 2 kb | 52.4 (13.5) | 151 | 41.0 (12.7) | | 101 |
|  | 5 kb | 52.8 (13.6) | 151 | 27.2 (8.5) | | 101 |
|  | 10 kb | 54.7 (14.1) | 151 | 67.9 (21.1) | | 101 |
| Total |  | 526.7 (136.1) |  | 425.7 (132.2) | |  |

**Table S2.** Statistics of the filtered raw sequences of pepper genomes.

| Species | Insert  Size | Raw data^a^ | Step1^b^ | Step2^c^ | Step3^d^ | Refined  data^e^ |
| --- | --- | --- | --- | --- | --- | --- |
| Baccatum | Single | NA | NA | NA | NA | 103.13 |
|  | 200bp | 221.09 | 220.80 | 185.14 | 142.44 | 4.70 |
|  | 400bp | 73.61 | 72.76 | 72.43 | 64.98 | 58.49 |
|  | 600bp | 72.06 | 68.81 | 67.51 | 64.29 | 46.86 |
|  | 2kb | 52.38 | 52.32 | 50.41 | 44.29 | 23.37 |
|  | 5kb | 52.82 | 52.75 | 48.03 | 41.72 | 22.17 |
|  | 10kb | 54.69 | 54.00 | 22.77 | 15.26 | 9.42 |
|  | Total | 526.65 Gb | 521.44 Gb | 446.29 Gb | 372.98 Gb | 268.14 Gb |
| Chinense | Single | NA | NA | NA | NA | 50.87 |
|  | 200bp | 98.88 | 98.64 | 96.65 | 72.14 | 25.50 |
|  | 400bp | 94.69 | 94.00 | 89.10 | 62.89 | 48.24 |
|  | 600bp | 96.05 | 95.75 | 87.55 | 68.79 | 61.12 |
|  | 2kb | 40.99 | 40.47 | 27.94 | 20.68 | 9.72 |
|  | 5b | 27.16 | 26.80 | 7.44 | 6.62 | 4.36 |
|  | 10kb | 67.86 | 67.68 | 32.43 | 21.53 | 13.93 |
|  | Total | 425.63 Gb | 423.34 Gb | 341.11 Gb | 252.65 Gb | 213.74 Gb |

^a^ Original raw data.

^b^ The remained sequences after filtration of bacterial genome.

^c^ The raw reads after removing duplicated reads.

^d^ The raw data after low quality trimming (<Q20).

^e^ The refined sequences after error correction and merging PE reads to single reads

**Table S3.** Summary of genome assemblies for *Capsicum* spp.

|  | Annuum^*^ | | | Baccatum | | | Chinense | | |
| --- | --- | --- | --- | --- | --- | --- | --- | --- | --- |
|  | Contig  (kbp) | | Scaffold  (Mbp) | Contig | | Scaffold | Contig | | Scaffold |
| N10 | 112.5 (1,927^th^) | | 7.4 (31^st^) | 129.8 (1,762^nd^) | | 6.0(40^th^) | 184.4 (1,146^th^) | | 9.5 (24^th^) |
| N20 | 76.0 (5,182^nd^) | | 5.0 (82^nd^) | 89.6 (4,691^st^) | | 4.2 (106^th^) | 122.1 (3,162^nd^) | | 7.4 (60^th^) |
| N30 | 55.0 (9,787^th^) | | 4.0 (151^st^) | 66.9 (8,733^rd^) | | 3.2 (194^th^) | 88.8 (6,037^th^) | | 5.7 (107^th^) |
| N40 | 40.6 (16,087^th^) | | 3.1 (240^th^) | 50.9 (14,068^th^) | | 2.6 (306^th^) | 67.0 (9,894^th^) | | 4.3 (168^th^) |
| N50 | 30.0 (24,618^th^) | | 2.5 (352^nd^) | 38.9 (21,059^th^) | | 2.0 (449^th^) | 50.3 (15,019^th^) | | 3.3 (247^th^) |
| N60 | 21.8 (36,237^th^) | | 1.9 (491^st^) | 29.0 (30,323^rd^) | | 1.6 (630^th^) | 37.1 (21,877^th^) | | 2.5 (353^rd^) |
| N70 | 15.3 (52,522^nd^) | | 1.5 (672^nd^) | 20.8 (42,920^th^) | | 1.2 (867^th^) | 26.4 (31,353^rd^) | | 1.8 (497^th^) |
| N80 | 9.8 (76,680^th^) | | 1.1 (915^th^) | 13.6 (61,249^th^) | | 0.8 (1,201^st^) | 17.1 (45,300^th^) | | 1.3 (695^th^) |
| N90 | 4.8 (119,071^st^) | | 0.6 (1,276^th^) | 6.8 (92,874^th^) | | 0.4 (1,748^th^) | 8.3 (69,622^nd^) | | 0.7 (1,015^th^) |
| Max | 442.1 | | 18.6 | 494 | | 16.4 | 872.2 | | 20.3 |
| Estimated genome size (Mbp) |  | | 3,480 |  | | 3,870 |  | | 3,213 |
| initial contig length (Mbp) |  | | 3,572 |  | | 3,831 |  | | 3,220 |
| Gap length (Mbp) | 99 | | | 110 | | | 41 | | |
| Number of assembly | 333,742 | 37,989 | | 250,070 | 25,349 | | 227,742 | 51,917 | |
| Total length  (Coverage) | 2.963 (85%) | | 3,063 (88%) | 3,104 (80%) | | 3,215 (83%) | 2,966(92%) | | 3,009 (94%) |

^*^ The pre-existing reference genome described in Kim *et al*[[4](#_ENREF_4)].

**Table S4.** Comparison of annotated gene models of the pepper genomes.

| Species | Version | Protein coding  Loci | Total CDS  Length (bp) | Ave CDS  Length (bp) |
| --- | --- | --- | --- | --- |
| Annuum | 2.0 | 35,884 | 39,133,866 | 1,091 |
| Baccatum | 1.2 | 35,874 | 40,376,406 | 1,125 |
| Chinense | 1.2 | 35,009 | 39,045,936 | 1,115 |

**Table S5.** BUSCO analysis with previous and current gene annotations of Annuum for assessment of gene coverage.

| Parameter^*^ | version 1.55 | | version 2.0 | |
| --- | --- | --- | --- | --- |
|  | Number | Percent (%) | Number | Percent (%) |
| Total BUSCO groups | 956 | 100% | 956 | 100% |
| Matched complete genes | 720 | 75.3% | 867 | 90.7% |
| Matched Fragmented genes | 89 | 9.3% | 31 | 3.2% |
| Missed genes | 147 | 15.4% | 58 | 6.1% |

^*^ Types of BUSCO database[[6](#_ENREF_6)] to compare gene coverage of previous and current gene models

**Table S6.** Statistics of twelve pseudomolecule chromosomes of the pepper genomes.

| Chr | Chromosome length (Mb) / number of anchored scaffolds | | |
| --- | --- | --- | --- |
|  | Annuum | Baccatum | Chinense |
| 1 | 309 / 229 | 259 / 189 | 241 / 143 |
| 2 | 170 / 125 | 169 / 126 | 170 / 122 |
| 3 | 283 / 350 | 298 / 209 | 275 / 174 |
| 4 | 240 / 156 | 219 / 168 | 232 / 121 |
| 5 | 239 / 163 | 228 / 169 | 237 / 99 |
| 6 | 242 / 173 | 253 / 157 | 251 / 129 |
| 7 | 251 / 173 | 255 / 210 | 127 / 234 |
| 8 | 142 / 52 | 210 / 158 | 196 / 113 |
| 9 | 271 / 196 | 197 / 153 | 258 / 144 |
| 10 | 233 / 170 | 230 / 179 | 233 / 122 |
| 11 | 267 / 188 | 264 / 179 | 251 / 141 |
| 12 | 251 / 224 | 237 / 186 | 226 / 122 |
| Total | 2,898 / 2,199 | 2,818/ 2,083 | 2,807 / 1,557 |

**Table S7.** Summary of generated pepper transcriptome data used in this study.

| Tissue | Description | Replicate | Baccatum | | Chinense | |
| --- | --- | --- | --- | --- | --- | --- |
|  |  |  | Number  of reads | Read  length | Number  of reads | Read  length |
| Leaf | Leaf, 6 week plant | 1^st^ | 15694816 | 101 | 13690578 | 101 |
|  |  | 2^nd^ | 14164792 | 101 | 13485006 | 101 |
|  |  | 3^rd^ | 15665678 | 101 | 21737894 | 101 |
| Root | Root, 6 week plant | 1^st^ | 13093384 | 101 | 15462240 | 101 |
|  |  | 2^nd^ | 18202358 | 101 | 12405602 | 101 |
|  |  | 3^rd^ | 12509506 | 101 | 12595874 | 101 |
| Stem | Stem, 6 week plant | 1^st^ | 27928864 | 126 | 20287050 | 101 |
|  |  | 2^nd^ | 12631034 | 126 | 13816800 | 101 |
|  |  | 3^rd^ | 30065376 | 126 | 15743552 | 101 |
| Flower | Flower | 1^st^ | 26745244 | 126 | 12811088 | 126 |
|  |  | 2^nd^ | 9007276 | 126 | 11502046 | 126 |
|  |  | 3^rd^ | NA | NA | 12498638 | 126 |
| PRMG | Pericarp of fruit, mature green (40DAP^*^) | 1^st^ | 11581676 | 126 | 14689406 | 151 |
|  |  | 2^nd^ | 12453072 | 126 | 22426788 | 151 |
|  |  | 3^rd^ | 11131000 | 126 | 16858578 | 151 |
| PLMG | Placenta of fruit, mature green (40DAP^*^) | 1^st^ | 11995022 | 126 | 7199654 | 151 |
|  |  | 2^nd^ | 12537818 | 126 | 7495938 | 151 |
|  |  | 3^rd^ | 13048646 | 126 | 7210916 | 151 |

^*^ means day after pollination

**Table S8.** Summary of validation for the assembled genomes using the transcriptome assemblies and filtered reads.

| Species | Data set^a^ | Total  number  (Length) | Average  (bp) | Analysis method^b^ | Identity  (%) | Coverage^c^ | | | |
| --- | --- | --- | --- | --- | --- | --- | --- | --- | --- |
|  |  |  |  |  |  | Matched | >70 % | >80 % | >90 % |
| Baccatum | Assembled  transcripts | 50,901 (42.21Mb) | 829.2 | Calculating query  (data) coverage  by BLASTN | 95 | 48,267 (94.8 %) | 48,063 (94.4 %) | 47,948 (94.2 %) | 47,452 (93.2 %) |
|  |  |  |  |  | 98 | 48,052 (94.4 %) | 47,762 (93.8 %) | 47,539 (93.4 %) | 46,684 (91.7 %) |
|  |  |  |  |  | 99 | 47,748 (93.8 %) | 47,179 (92.7 %) | 46,585 (91.5 %) | 44,747 (87.9 %) |
|  | Single | 544 M  (103.1 Gb) | 189.5 | Calculating read mapping  coverage | 98 | 543 M (99.7 %) | - | - | - |
|  | PE | 749 M  (110.0 Gb) | 146.9 |  |  | 748 M (99.8 %) | - | - | - |
|  | Total | 1,293 (213.1) | 164.8 |  |  | 1,291 M  (99.8 %) | - | - | - |
| Chinense | Assembled  transcripts | 48,089 (40.05 Mb) |  | Calculating query (data) coverage by BLASTN | 95 | 46,214 (96.1 %) | 46,014 (95.7 %) | 45,919 (95.5 %) | 45,493 (94.6 %) |
|  |  |  | 832.86 |  | 98 | 45,981 (95.6 %) | 45,655 (94.9 %) | 45,433 (94.5 %) | 44,647 (92.3 %) |
|  |  |  |  |  | 99 | 45,678 (95.0 %) | 45,058 (93.7 %) | 44,489 (92.5 %) | 42,753 (88.9 %) |
|  | Single | 329 M (50.9 Gb) | 154.6 | Calculating read mapping coverage | 98 | 322 M (97.7 %) | - | - | - |
|  | PE | 1,356 M (134.8 Gb) | 99.4 |  |  | 1,331 M (98.2 %) | - | - | - |
|  | Total | 1,685 M (185.7 Gb) | 110.2 |  |  | 1,653 M (98.1 %) |  |  |  |

^a^ Data types for validation. The *de novo* transcriptome assemblies were constructed by Velvet[[7](#_ENREF_7)] and Oases[[8](#_ENREF_8)]

pipeline (k=91 for Baccatum Chinense) using integrated transcriptome data described in Table S7. Single and PE mean merged and not merged paired-end data, respectively.

^b^ Comparison method between the assembled genomes as query sequences and the data set. The assembled transcripts were matched using BLASTN considering various similarity and query coverage. Single and PE reads were mapped using CLC Assembly Cell (CLC Bio, Denmark) considering 98% similarity and >50% read fraction.

^c^ The matched coverage of the assembled transcripts and the filtered raw sequences.

**Table S9.** Amount of repeat sequences in the assembled genomes and initial contig sets.

| Type of TE | Annuum | | | | | Baccatum | | | | Chinense | | | |
| --- | --- | --- | --- | --- | --- | --- | --- | --- | --- | --- | --- | --- | --- |
|  | Assembly | | | Initial contig | | Assembly | | Initial contig | | Assembly | | Initial contig | |
|  | Length  (Mbp) | Ratio  (%) |  | Length | Ratio | Length | Ratio | Length | Ratio | Length | Ratio | Length | Ratio |
| DNA elements | 291 | 9.5 |  | 382 | 10.7 | 307 | 9.5 | 381 | 9.9 | 305 | 10.1 | 340 | 10.6 |
| LINE elements | 66 | 2.2 |  | 78 | 2.2 | 68 | 2.1 | 79 | 2.1 | 69 | 2.3 | 73 | 2.3 |
| SINE elements | 14 | 0.5 |  | 25 | 0.7 | 19 | 0.6 | 33 | 0.9 | 19 | 0.6 | 24 | 0.7 |
| LTR/Gypsy | 1827 | 59.6 |  | 2107 | 59.0 | 1929 | 60.0 | 2370 | 61.9 | 1762 | 58.6 | 1910 | 59.3 |
| LTR/Copia | 182 | 5.9 |  | 214 | 6.0 | 179 | 5.6 | 201 | 5.2 | 170 | 5.6 | 195 | 6.1 |
| LTR/  Caulimoviridae | 65 | 2.1 |  | 129 | 3.6 | 68 | 2.1 | 116 | 3.0 | 66 | 2.2 | 108 | 3.4 |
| rDNA | 2 | 0.1 |  | 4 | 0.1 | 26 | 0.8 | 56 | 1.5 | 2 | 0.1 | 5 | 0.2 |
| Simple repeat | 36 | 1.2 |  | 54 | 1.5 | 37 | 1.2 | 50 | 1.3 | 37 | 1.2 | 46 | 1.4 |
| Others | 11 | 0.4 |  | 19 | 0.5 | 13 | 0.4 | 18 | 0.5 | 9 | 0.3 | 19 | 0.6 |
| Total | 2495 | 81.5 |  | 3012 | 84.3 | 2647 | 82.3 | 3304 | 86.2 | 2438 | 81.0 | 2720 | 84.5 |

**Table S10.** Statistics of subgroups in the *gypsy* superfamily.

| Type of TE | Annuum | | | | | Baccatum | | | | Chinense | | | |
| --- | --- | --- | --- | --- | --- | --- | --- | --- | --- | --- | --- | --- | --- |
|  | Genome | | | Initial contig | | Genome | | Initial contig | | Genome | | Initial contig | |
|  | Length  (Mbp) | Ratio  (%) |  | Length | Ratio | Length | Ratio | Length | Ratio | Length | Ratio | Length | Ratio |
| Athila | 278 | 9.1 |  | 304 | 8.5 | 460 | 14.3 | 676 | 17.7 | 276 | 9.2 | 278 | 8.6 |
| Crm | 44 | 1.4 |  | 55 | 1.5 | 48 | 1.5 | 86 | 2.2 | 45 | 1.5 | 50 | 1.5 |
| Del | 1291 | 42.1 |  | 1482 | 41.5 | 1201 | 37.4 | 1337 | 34.9 | 1242 | 41.3 | 1343 | 41.7 |
| Galadriel | 1 | 0.0 |  | 2 | 0.0 | 2 | 0.1 | 2 | 0.1 | 1 | 0.0 | 1 | 0.0 |
| Reina | 3 | 0.1 |  | 3 | 0.1 | 3 | 0.1 | 3 | 0.1 | 3 | 0.1 | 3 | 0.1 |
| Tat | 142 | 4.6 |  | 187 | 5.2 | 153 | 4.8 | 192 | 5.0 | 142 | 4.7 | 159 | 4.9 |
| Others | 67 | 2.2 |  | 76 | 2.1 | 62 | 1.9 | 74 | 1.9 | 54 | 1.8 | 76 | 2.3 |
| Total | 1827 | 59.6 |  | 2107 | 59.0 | 1929 | 60.0 | 2370 | 61.9 | 1762 | 58.6 | 1910 | 59.3 |

**Table S11.** Protein data information used in this study.

| Species | Version | Number  of genes | Download URLs |
| --- | --- | --- | --- |
| *C. annuum* | v2.0 | 35,884 | http://peppergenome.snu.ac.kr/ |
| *C. baccatum* | v1.2 | 35,874 | http://peppergenome.snu.ac.kr/ |
| *C. chinense* | v1.2 | 35,009 | http://peppergenome.snu.ac.kr/ |
| *Solanum lycopersicum* | v2.3 | 34,725 | http://solgenomics.net/ |
| *Solanum tuberosum* | PGSC v3.4 | 39,028 | http://www.potatogenome.net/ |
| *Arabidopsis thaliana* | TAIR10 | 27,416 | http://www.arabidopsis.org/ |
| *Vitis vinifera* | v2.0 | 26,346 | http://www.plantgdb.org/VvGDB/ |
| *Oryza sativa* | MSU RAGP7 | 39,045^*^ | http://rice.plantbiology.msu.edu/ |

^*^ The number of non-TEs related genes in rice

**Table S12.** Statistics of intact LTR retrotransposons in three pepper genomes.

| Superfamily | Subgroup | Annuum | Baccatum | Chinense |
| --- | --- | --- | --- | --- |
| Gypsy | *del* | 45,588 | 39,650 | 44,151 |
|  | *athila* | 9,311 | 20,945 | 9,057 |
|  | *crm* | 2,157 | 2,538 | 2,145 |
|  | *tat* | 2,091 | 2,149 | 2,084 |
|  | *reina* | 585 | 527 | 551 |
|  | *galadriel* | 117 | 120 | 111 |
|  | *others* | 24,049 | 23,110 | 23,239 |
| Copia | *tork* | 5,082 | 5,276 | 4,894 |
|  | *retrofit* | 2,606 | 3,126 | 2,642 |
|  | *sire* | 2,130 | 1,860 | 2,046 |
|  | *oryco* | 1,233 | 1,069 | 1,193 |
|  | *others* | 2,110 | 1,389 | 1,416 |
| Caulimoviridae | *cavemovirus* | 866 | 1,131 | 1,057 |
|  | *caulimovirus* | 216 | 218 | 167 |
|  | *badnavirus* | 194 | 268 | 214 |
|  | *soymovirus* | 15 | 7 | 18 |
|  | others | 308 | 258 | 333 |
| Total | Total | 98,658 | 103,641 | 95,318 |

**Table S13.** Classification of whole (retroduplicated, normal) NLR gene family in 6 plant genomes.

|  | Total (Retroduplicated, normal) | | | | | |
| --- | --- | --- | --- | --- | --- | --- |
| Subgroup | Annuum | Baccatum | Chinense | Tomato | Potato | Rice |
| TNL | 64 (0,61) | 40 (0,40) | 48 (2,41) | 33 (2,30) | 64 (4,55) | 1 (0,1) |
| CNL-G1 | 126 (5,119) | 149 (11,135) | 109 (3,105) | 29 (4,22) | 36 (8,27) | 0 (0) |
| CNL-G2 | 194 (78,68) | 221 (83,86) | 154 (59,59) | 4 (0,4) | 1 (0,1) | 0 (0) |
| CNL-G3 | 43 (0,42) | 36 (1,33) | 45 (0,45) | 19 (2,16) | 18 (3,15) | 0 (0) |
| CNL-G4 | 58 (4,50) | 59 (5,51) | 50 (5,45) | 17 (4,12) | 27 (7,18) | 32 (3,27) |
| CNL-G5 | 21 (0,21) | 21 (1,20) | 18 (1,17) | 24 (2,21) | 35 (9,22) | 10 (0,10) |
| CNL-G6 | 57 (7,43) | 70 (8,52) | 52 (5,44) | 16 (2,14) | 41 (10,26) | 0 (0) |
| CNL-G7 | 41 (0,40) | 40 (0,40) | 29 (0,29) | 16 (0,16) | 31 (3,26) | 167 (7,155) |
| CNL-G8 | 16 (0,15) | 17 (1,16) | 18 (0,18) | 10 (0,10) | 17 (1,15) | 0 (0) |
| CNL-G9 | 72 (3,65) | 69 (2,66) | 69 (2,65) | 33 (1,31) | 55 (18,32) | 2 (0,2) |
| CNL-G10 | 80 (5,74) | 98 (9,86) | 83 (8,70) | 30 (0,30) | 41 (7,30) | 17 (0,17) |
| CNL-G11 | 11 (1,10) | 8 (1,7) | 10 (0,9) | 5 (2,3) | 12 (5,7) | 0 (0) |
| CNL-G12 | 19 (1,18) | 16 (1,15) | 14 (0,14) | 2 (0,2) | 16 (1,14) | 0 (0) |
| CNL-G13 | 1 (0,1) | 4 (0,4) | 3 (0,3) | 12 (2,10) | 25 (3,21) | 3 (0,3) |
| CNL-G14 | 5 (0,5) | 6 (0,6) | 7 (0,7) | 4 (0,4) | 7 (0,7) | 43 (2,41) |
| CNL-Monocot1 | 0 (0,0) | 0 (0) | 0 (0) | 0 (0) | 0 (0) | 206 (8,194) |
| CNL-Monocot2 | 0 (0,0) | 0 (0) | 0 (0) | 0 (0) | 0 (0) | 34 (7,25) |
| Unclassified | 27 (1,25) | 29 (0,28) | 27 (1,26) | 13 (0,11) | 17 (2,15) | 12 (0,12) |
| Total | 835 (105,657) | 883 (123,685) | 736 (86,597) | 267 (21,236) | 443 (81,331) | 527 (27,487) |

**Table S14.** List of retroduplicated NLR genes in the *Capsicum* spp.

See separate excel file.

**Table S15.** Statistics of LTR-Rs capturing NLR gene family.

| Type | Total | | | | CNL-G2 | | |
| --- | --- | --- | --- | --- | --- | --- | --- |
|  | Annuum | | Baccatum | Chinense | Annuum | Baccatum | Chinense |
| Gypsy | | 12 | 5 | 4 | 10 | 5 | 4 |
| Copia | | 17 | 31 | 16 | 15 | 23 | 14 |
| Caulimoviridae | | 0 | 1 | 1 |  | 1 | 1 |
| Potential non-autonomous family | | 76 | 86 | 65 | 53 | 54 | 40 |
| Total | | 105 | 123 | 86 | 78 | 83 | 59 |

**Table S16.** Number of intron-containing NLRs inside LTR-Rs.

|  | Total | | | | | |
| --- | --- | --- | --- | --- | --- | --- |
| Subgroup | Annuum | Baccatum | Chinense | Tomato | Potato | Rice |
| TNL | 0 | 0 | 2 | 1 | 3 | 0 |
| CNL-G1 | 1 | 5 | 1 | 2 | 3 | 0 |
| CNL-G2 | 25 | 22 | 18 | 0 | 0 | 0 |
| CNL-G3 | 0 | 0 | 0 | 2 | 3 | 0 |
| CNL-G4 | 2 | 0 | 2 | 1 | 3 | 1 |
| CNL-G5 | 0 | 0 | 0 | 0 | 1 | 0 |
| CNL-G6 | 2 | 4 | 1 | 0 | 2 | 0 |
| CNL-G7 | 0 | 0 | 0 | 0 | 2 | 3 |
| CNL-G8 | 0 | 1 | 0 | 0 | 1 | 0 |
| CNL-G9 | 0 | 0 | 1 | 1 | 8 | 0 |
| CNL-G10 | 3 | 5 | 4 | 0 | 4 | 0 |
| CNL-G11 | 0 | 0 | 0 | 1 | 1 | 0 |
| CNL-G12 | 0 | 0 | 0 | 0 | 0 | 0 |
| CNL-G13 | 0 | 0 | 0 | 2 | 1 | 0 |
| CNL-G14 | 0 | 0 | 0 | 0 | 0 | 1 |
| CNL-Monocot1 | 0 | 0 | 0 | 0 | 0 | 7 |
| CNL-Monocot2 | 0 | 0 | 0 | 0 | 0 | 1 |
| Unclassified | 1 | 0 | 0 | 0 | 0 | 0 |
| Total | 34 | 37 | 29 | 10 | 32 | 13 |

**Table S17.** Number of retroduplicated NLRs having reduced number of introns comparing to their parental sequences.

|  | Total | | | | | |
| --- | --- | --- | --- | --- | --- | --- |
| Subgroup | Annuum | Baccatum | Chinense | Tomato | Potato | Rice |
| TNL | 0 | 0 | 2 | 1 | 3 | 0 |
| CNL-G1 | 5 | 8 | 2 | 3 | 6 | 0 |
| CNL-G2 | 66 | 74 | 55 | 0 | 0 | 0 |
| CNL-G3 | 0 | 0 | 0 | 2 | 3 | 0 |
| CNL-G4 | 4 | 5 | 5 | 3 | 7 | 2 |
| CNL-G5 | 0 | 1 | 0 | 2 | 9 | 0 |
| CNL-G6 | 4 | 5 | 2 | 2 | 9 | 0 |
| CNL-G7 | 0 | 0 | 0 | 0 | 2 | 3 |
| CNL-G8 | 0 | 1 | 0 | 0 | 1 | 0 |
| CNL-G9 | 2 | 1 | 1 | 0 | 17 | 0 |
| CNL-G10 | 4 | 8 | 8 | 0 | 6 | 0 |
| CNL-G11 | 0 | 1 | 0 | 1 | 4 | 0 |
| CNL-G12 | 1 | 0 | 0 | 0 | 1 | 0 |
| CNL-G13 | 0 | 0 | 0 | 0 | 2 | 0 |
| CNL-G14 | 0 | 0 | 0 | 0 | 0 | 0 |
| CNL-Monocot1 | 0 | 0 | 0 | 0 | 0 | 7 |
| CNL-Monocot2 | 0 | 0 | 0 | 0 | 0 | 4 |
| Unclassified | 1 | 0 | 0 | 0 | 1 | 0 |
| Total | 87 | 104 | 75 | 14 | 71 | 16 |

**Table S18.** Number of whole genes inside LTR-Rs in 6 plant genomes.

| Description | Annuum | Baccatum | Chinense | Tomato | Potato | Rice |
| --- | --- | --- | --- | --- | --- | --- |
| Leucine rich repeat | 67 | 71 | 63 | 13 | 55 | 10 |
| Protein kinase domain | 70 | 48 | 45 | 23 | 51 | 34 |
| PPR repeat | 17 | 36 | 14 | 6 | 14 | 4 |
| SRF-type transcription factor | 37 | 72 | 22 | 14 | 30 | 2 |
| F-box domain | 29 | 37 | 22 | 16 | 44 | 8 |
| Auxin responsive protein | 26 | 28 | 24 | 22 | 42 | 2 |
| Cytochrome P450 | 19 | 30 | 30 | 21 | 82 | 19 |
| Genes containing functional domains^a^ | 1213 | 1308 | 1272 | 812 | 1541 | 619 |
| Expressed genes^b^ | 1022 | 1444 | 903 | 740 | 1573 | 667 |
| Total retrogenes  (% of total genes) | 2289 (6%) | 3632 (10%) | 2071 (6%) | 1878 (5%) | 3898 (10%) | 1398 (4%) |

^a^ The genes containing domains predicted by Pfam v27.0 in InterproScan v5.15-54.0[[9](#_ENREF_9)].

^b^ The genes expressed in one or more tissues of RNA-Seq described in Table S7 and previous analyses[[4](#_ENREF_4), [10-12](#_ENREF_10)].

**Table S19.** Location of synteny blocks including *R3a*, *I2* and *L* in chromosome 11 of Solanaceae genomes.

| **MarkerID**^*^ | **Potato** | **Tomato** | **Pepper** |
| --- | --- | --- | --- |
| CT120 | NA | 52,949,988 | 239,487,777 |
| cLET24J2A | 41,027,873 | 52,557,251 | NA |
| TG26 | 40,998,682 | 52,533,343 | 247,744,464 |
| *R3a* | NA | NA | NA |
| TAO1 | 40,200,818 | 52,113,146 | 251,893,617 |
| TG105 | 40,168,487 | 52,067,537 | 252,637,418 |
| *I2* | NA | NA | NA |
| cLET5E4 | 38,769,809 | 51,189,476 | 262,852,256 |
| *L* | NA | NA | 263,643,298 |

^*^ Marker IDs described in Huang et al[[5](#_ENREF_5)] and gene names.

**Table S20.** Candidates of parent sequences of *L* genes in assembled pepper genomes.

| Species | Index | Parent sequence information (chromosome / start / end position) | Alignment  coverage^a^ | Similarity^b^ | *Ks* value^c^ | Duplication  time (MYA) ^d^ |
| --- | --- | --- | --- | --- | --- | --- |
| Annuum | P1 | 11 / 265700158 bp / 265721697 bp | 100% | 83.06% | 0.124 | 8.91 |
| Baccatum | P2 | scaffold2911 / 9308 bp / 13070 bp | 95.93% | 76.14% | 0.138 | 9.91 |
| Baccatum | P3 | 11 / 253166172 bp / 253180656 bp | 95.93% | 79.80% | 0.143 | 10.27 |
| Baccatum | P4 | 11 / 259281766 bp / 259329615 bp | 100% | 79.80% | 0.154 | 11.06 |
| Chinense | P5 | 11 / 241838842 bp / 241841806 bp | 73.72% | 83.15% | 0.153 | 10.99 |
| Chinense | P6 | 11 / 250025753 bp / 250043182 bp | 100% | 77.50% | 0.174 | 12.50 |

^a^ indicates query coverage of parental sequences for *L* gene.

^b^ means identity calculated using BLASTP between parental sequences and *L* genes.

^c^ indicates *Ks* values (synonymous substitutions/synonymous site) between parental sequences and *L* genes.

^d^ indicates the duplication time between parental sequences and *L* estimated using the formula, *Ks* value / 2r, (r = 6.96 × 10^−9^) as described in the Method.

**Table S21.** Position of sequence mutation among L genes in *Capsicum* species.

| Position | *C. annuum* | *C. chinense* | *C. baccatum* | *C. chacoense* |
| --- | --- | --- | --- | --- |
| 73 | F | F | L | L |
| 89 | L | L | Q | Q |
| 97 | L | R | L | L |
| 110 | S | S | N | N |
| 117 | L | R | L | L |
| 164 | T | T | S | T |
| 232 | D | D | G | G |
| 245 | S | A | A | A |
| 335 | E | E | K | K |
| 360 | L | P | P | P |
| 368 | Q | R | Q | Q |
| 420 | L | L | M | M |
| 423 | T | N | N | N |
| 431 | R | Q | R | R |
| 444 | Q | K | K | K |
| 613 | R | Q | R | R |
| 777 | R | Q | Q | Q |
| 829 | T | T | M | M |
| 844 | S | S | S | T |
| 864 | N | I | N | N |
| 887 | K | E | E | E |
| 923 | S | S | F | F |
| 950 | T | I | T | T |
| 971 | E | E | A | E |
| 995 | I | I | V | V |
| 998 | P | P | T | T |
| 1041 | V | V | A | A |
| 1077 | G | W | W | W |
| 1108 | G | G | S | S |
| 1115 | H | Q | Q | Q |
| 1126 | N | N | H | R |
| 1144 | C | C | F | F |
| 1150 | V | V | T | T |
| 1152 | V | V | D | D |
| 1170 | S | S | S | T |
| 1173 | I | I | F | F |
| 1175 | K | N | N | N |
| 1180 | Q | R | R | R |
| 1236 | S | C | S | S |
| 1289 | Y | Y | C | C |
| 1308 | E | K | E | E |
| 1314 | K | E | E | E |

**Table S22.** Duplication history of *R3a* and its homologs.

| **Group** | ***Ks* value** | **Duplication time (MYA)** | **Duplication pair (chromosome / start / end position) ^*^** |
| --- | --- | --- | --- |
| G1 | 0.0802294 | 5.76 | I2GA_SH23_1,R3a |
| G2 | 0.0941522 | 6.76 | I2GA_SH194_2,I2GA_SH23_3 |
| G3 | 0.111595 | 8.02 | G1,G2 |
| G4 | 0.135944 | 9.77 | chr11_39928166_39949970,G3 |
| G5 | 0.14403 | 10.35 | chr11_39938386_40018926,G4 |
| G6 | 0.160086 | 11.50 | chr11_39898603_39931945,G5 |
| G7 | 0.353544 | 25.40 | chr11_41310964_41368788,G6 |

* indicates duplicated gene pair and gene names are originated from Huang *et al*[[5](#_ENREF_5)]. Each duplication pair was determined via single linkage clustering analyses using *Ks* values among *R3a* and its homologs.

**Table S23.** List of NLR gene in sequence block containing anthracnose resistance genes for *C. capsici* in chromosome 3 of *C. baccatum*.

| Gene_ID | Type of LTR-Rs  capturing genes | Start possition  (bp) | Subgroup | Retorgene^a^ | Conservativeness | Expression (RPKM) | | | | | | Duplication  time (MYA) |
| --- | --- | --- | --- | --- | --- | --- | --- | --- | --- | --- | --- | --- |
|  |  |  |  |  |  | Leaf | Stem | Root | Flower | PLMG | PRMG |  |
| CB.CBv1.2.scaffold2141.7 | Copia | 64071960 | G2 | Y | Both | 0.15 | 0.00 | 0.00 | 0.00 | 0.00 | 0.00 | 0 |
| CB.CBv1.2.scaffold778.8 | NA | 64297009 | G2 | N | Both | 0.00 | 0.57 | 0.91 | 0.23 | 0.78 | 0.09 | 0 |
| CB.CBv1.2.scaffold778.21 | Copia | 64422917 | G2 | Y | N | 2.18 | 2.76 | 0.20 | 0.89 | 2.09 | 4.37 | 0 |
| CB.CBv1.2.scaffold1022.13 | Gypsy | 61592098 | G2 | Y | N | 9.30 | 7.89 | 1.88 | 5.32 | 9.43 | 9.57 | 0 |
| CB.CBv1.2.scaffold1022.3 | Non-autonomous | 61274829 | G2 | Y | CA | 0.00 | 0.39 | 2.56 | 0.00 | 0.18 | 0.08 | 0.02 |
| CB.CBv1.2.scaffold778.3 | NA | 64256781 | G2 | N | N | 0.06 | 0.21 | 0.00 | 0.19 | 0.25 | 0.00 | 0.19 |
| CB.CBv1.2.scaffold778.16 | Non-autonomous | 64381670 | G2 | Y | N | 0.70 | 0.45 | 0.10 | 0.45 | 0.43 | 0.64 | 0.32 |
| CB.CBv1.2.scaffold778.12 | Copia | 64349177 | G2 | Y | Both | 3.88 | 3.70 | 0.53 | 0.99 | 4.18 | 5.20 | 0.32 |
| CB.CBv1.2.scaffold778.13 | NA | 64353012 | G2 | N | N | 1.55 | 1.73 | 0.41 | 0.23 | 1.55 | 1.80 | 0.32 |
| CB.CBv1.2.scaffold2141.12 | NA | 64182211 | G2 | N | CA | 0.00 | 0.00 | 0.52 | 0.00 | 0.00 | 0.00 | 0.55 |
| CB.CBv1.2.scaffold778.28 | Copia | 64481535 | G2 | Y | Both | 0.05 | 0.00 | 1.70 | 0.00 | 0.00 | 0.00 | 0.77 |
| CB.CBv1.2.scaffold1022.20 | NA | 61672870 | G2 | N | N | 1.63 | 1.48 | 1.63 | 0.56 | 2.42 | 2.81 | 0.83 |
| CB.CBv1.2.scaffold1022.27 | Gypsy | 61751646 | G2 | Y | CC | 6.30 | 5.10 | 5.69 | 3.84 | 5.85 | 9.19 | 0.90 |
| CB.CBv1.2.scaffold778.4 | NA | 64260860 | G2 | N | N | 1.31 | 2.13 | 2.16 | 0.72 | 2.22 | 0.48 | 0.96 |
| CB.CBv1.2.scaffold1022.30 | NA | 61799418 | G2 | N | N | 0.79 | 0.84 | 0.40 | 0.64 | 0.69 | 0.79 | 1.01 |
| CB.CBv1.2.scaffold2141.4 | NA | 64001434 | G2 | N | N | 0.06 | 0.41 | 2.29 | 0.00 | 0.54 | 0.00 | 1.01 |
| CB.CBv1.2.scaffold1022.21 | Non-autonomous | 61702188 | G2 | Y | Both | 1.76 | 2.19 | 13.43 | 1.15 | 1.41 | 0.56 | 1.14 |
| CB.CBv1.2.scaffold778.30 | Gypsy | 64516490 | G2 | Y | Both | 2.15 | 4.44 | 0.42 | 0.78 | 5.04 | 5.83 | 1.24 |
| CB.CBv1.2.scaffold778.18 | Non-autonomous | 64403694 | G2 | Y | Both | 1.83 | 1.64 | 0.79 | 1.41 | 3.08 | 0.26 | 1.31 |
| CB.CBv1.2.scaffold2141.8 | NA | 64073030 | G2 | N | N | 0.05 | 0.00 | 0.00 | 0.00 | 0.07 | 0.12 | 1.45 |
| CB.CBv1.2.scaffold778.26 | NA | 64465666 | G2 | N | N | 0.00 | 0.00 | 1.16 | 0.00 | 0.00 | 0.00 | 1.45 |
| CB.CBv1.2.scaffold778.11 | NA | 64323895 | G2 | N | N | 1.13 | 1.10 | 6.57 | 1.18 | 2.92 | 0.13 | 1.47 |
| CB.CBv1.2.scaffold778.5 | Non-autonomous | 64270559 | G2 | Y | N | 0.18 | 0.09 | 0.06 | 0.00 | 0.00 | 0.00 | 1.47 |
| CB.CBv1.2.scaffold778.14 | NA | 64360975 | G2 | N | N | 0.73 | 0.62 | 4.01 | 0.29 | 0.16 | 0.26 | 1.49 |
| CB.CBv1.2.scaffold1022.31 | Non-autonomous | 61808602 | G2 | Y | Both | 3.51 | 5.80 | 3.59 | 3.90 | 8.07 | 4.82 | 1.51 |
| CB.CBv1.2.scaffold778.20 | NA | 64413282 | G2 | N | Both | 5.43 | 3.90 | 0.70 | 2.17 | 5.13 | 8.60 | 1.82 |
| CB.CBv1.2.scaffold778.10 | Non-autonomous | 64322043 | G2 | Y | N | 0.00 | 0.00 | 0.00 | 0.00 | 0.00 | 0.00 | 1.97 |
| CB.CBv1.2.scaffold778.2 | Copia | 64245846 | G2 | Y | Both | 0.99 | 0.84 | 2.66 | 0.93 | 1.84 | 1.86 | 2.07 |
| CB.CBv1.2.scaffold2141.13 | NA | 64186603 | G2 | N | Both | 0.00 | 0.00 | 0.00 | 0.00 | 0.00 | 0.00 | 2.42 |
| CB.CBv1.2.scaffold1022.32 | NA | 61824638 | G12 | N | N | 0.36 | 0.31 | 3.85 | 0.23 | 0.46 | 0.28 | 2.64 |
| CB.CBv1.2.scaffold1022.33 | NA | 61828481 | G12 | N | N | 0.19 | 0.54 | 0.94 | 0.00 | 0.83 | 0.30 | 2.64 |
| CB.CBv1.2.scaffold1524.8 | NA | 61124572 | G10 | N | CA | 4.46 | 6.34 | 6.45 | 2.68 | 7.88 | 3.60 | 2.64 |
| CB.CBv1.2.scaffold2141.5 | NA | 64010653 | G2 | N | N | 0.00 | 0.00 | 0.00 | 0.00 | 0.00 | 0.00 | 3.16 |
| CB.CBv1.2.scaffold778.24 | NA | 64443560 | G2 | N | N | 0.11 | 0.00 | 0.06 | 0.00 | 0.00 | 0.00 | 3.52 |
| CB.CBv1.2.scaffold778.7 | Non-autonomous | 64279281 | G2 | Y | Both | 0.84 | 3.98 | 0.99 | 1.66 | 1.72 | 1.78 | 4.18 |
| CB.CBv1.2.scaffold1524.6 | NA | 61160493 | G10 | N | CA | 21.06 | 9.76 | 1.82 | 2.83 | 15.89 | 20.61 | 4.44 |
| CB.CBv1.2.scaffold1022.23 | NA | 61712836 | G2 | N | Both | 3.97 | 4.20 | 0.62 | 0.90 | 5.34 | 8.17 | 4.71 |
| CB.CBv1.2.scaffold1524.32 | NA | 60790430 | G10 | N | CC | 6.11 | 3.17 | 1.72 | 3.12 | 6.37 | 5.72 | 5.55 |
| CB.CBv1.2.scaffold1022.1 | Copia | 61227426 | G2 | Y | CA | 0.12 | 0.75 | 1.12 | 0.13 | 0.26 | 0.00 | 5.58 |
| CB.CBv1.2.scaffold2141.10 | NA | 64143946 | G2 | N | N | 0.16 | 0.19 | 0.44 | 0.00 | 0.12 | 0.38 | 5.79 |
| CB.CBv1.2.scaffold1524.14 | Copia | 60995617 | G10 | Y | N | 0.30 | 0.45 | 0.75 | 0.00 | 0.72 | 0.17 | 6.09 |
| CB.CBv1.2.scaffold1524.15 | NA | 60970798 | G10 | N | Both | 0.00 | 0.00 | 0.00 | 0.00 | 0.00 | 0.00 | 6.21 |
| CB.CBv1.2.scaffold1524.12 | NA | 61044887 | G10 | N | CC | 2.93 | 7.58 | 16.42 | 2.69 | 5.93 | 1.30 | 6.21 |
| CB.CBv1.2.scaffold1524.33 | NA | 60765379 | G10 | N | CA | 0.53 | 0.34 | 7.06 | 0.11 | 0.28 | 0.00 | 6.35 |
| CB.CBv1.2.scaffold2141.9 | NA | 64091925 | G2 | N | N | 0.00 | 0.00 | 0.05 | 0.00 | 0.00 | 0.00 | 6.42 |
| CB.CBv1.2.scaffold1022.34 | NA | 61835735 | G12 | N | N | 3.43 | 3.16 | 5.96 | 2.06 | 3.56 | 5.68 | 6.78 |
| CB.CBv1.2.scaffold2141.1 | Non-autonomous | 63969541 | G2 | Y | N | 0.99 | 2.94 | 7.40 | 0.34 | 5.34 | 0.90 | 7.60 |
| CB.CBv1.2.scaffold1022.14 | Copia | 61612938 | G2 | Y | N | 0.27 | 0.24 | 1.87 | 0.00 | 2.46 | 1.86 | 7.78 |
| CB.CBv1.2.scaffold1524.34 | NA | 60732231 | G10 | N | N | 0.99 | 6.49 | 6.69 | 1.12 | 6.69 | 2.15 | 7.93 |
| CB.CBv1.2.scaffold1524.11 | NA | 61089342 | G10 | N | CA | 2.72 | 2.11 | 84.87 | 0.21 | 13.96 | 0.30 | 8.17 |
| CB.CBv1.2.scaffold2141.3 | NA | 63993536 | G2 | N | N | 0.33 | 0.96 | 35.37 | 0.10 | 0.68 | 0.12 | 8.22 |
| CB.CBv1.2.scaffold2141.14 | Copia | 64195096 | G2 | Y | N | 0.00 | 0.04 | 14.93 | 0.00 | 0.00 | 0.00 | 8.73 |
| CB.CBv1.2.scaffold1022.25 | Non-autonomous | 61739170 | G2 | Y | N | 0.00 | 0.00 | 3.19 | 0.00 | 0.00 | 0.00 | 9.56 |
| CB.CBv1.2.scaffold1524.35 | NA | 60712633 | G10 | N | N | 0.00 | 0.00 | 0.09 | 0.20 | 0.05 | 0.04 | 12.35 |
| CB.CBv1.2.scaffold1524.23 | Non-autonomous | 60850721 | G10 | Y | N | 1.07 | 1.92 | 1.51 | 1.75 | 1.75 | 0.71 | 16.53 |
| CB.CBv1.2.scaffold1524.22 | NA | 60864899 | G10 | N | CC | 1.05 | 1.07 | 3.81 | 1.45 | 1.53 | 1.09 | 16.53 |
| CB.CBv1.2.scaffold1524.30 | Non-autonomous | 60802398 | G10 | Y | CC | 0.65 | 3.14 | 2.95 | 0.25 | 6.35 | 6.47 | 20.08 |
| CB.CBv1.2.scaffold544.12 | NA | 62372964 | G12 | N | Both | 3.61 | 1.35 | 2.27 | 1.02 | 0.43 | 0.28 | 29.67 |
| CB.CBv1.2.scaffold2141.11 | Non-autonomous | 64152036 | G2 | Y | Both | 0.00 | 0.24 | 1.40 | 0.00 | 0.00 | 0.00 | NA |
| CB.CBv1.2.scaffold1524.9 | Non-autonomous | 61115279 | G10 | Y | N | 0.19 | 0.00 | 0.40 | 0.00 | 0.00 | 0.00 | NA |
| CB.CBv1.2.scaffold778.23 | Non-autonomous | 64436558 | G2 | Y | N | 0.00 | 0.00 | 0.00 | 0.00 | 0.00 | 0.00 | NA |
| CB.CBv1.2.scaffold778.22 | NA | 64434235 | G2 | N | N | 0.00 | 0.00 | 0.00 | 0.00 | 0.00 | 0.00 | NA |
| CB.CBv1.2.scaffold1022.28 | NA | 61764084 | G2 | N | N | 0.00 | 0.00 | 0.00 | 0.00 | 0.00 | 0.23 | NA |

^a^ indicates retroduplicated (Y) or normal (N) genes listed in Additional file 2 (Table S14).

**References**

1 Kim S, Kim MS, Kim YM, Yeom SI, Cheong K, Kim KT, et al. Integrative structural annotation of *de novo* RNA-Seq provides an accurate reference gene set of the enormous genome of the onion (*Allium cepa* L.). DNA Res. 2015;22:19-27.

2 Haas BJ, Salzberg SL, Zhu W, Pertea M, Allen JE, Orvis J, et al. Automated eukaryotic gene structure annotation using EVidenceModeler and the program to assemble spliced alignments. Genome Biol. 2008;9:R7.

3 Stanke M, Tzvetkova A, Morgenstern B. AUGUSTUS at EGASP: using EST, protein and genomic alignments for improved gene prediction in the human genome. Genome Biol. 2006;7:S11-8.

4 Kim S, Park M, Yeom SI, Kim YM, Lee JM, Lee HA, et al. Genome sequence of the hot pepper provides insights into the evolution of pungency in *Capsicum* species. Nat Genet. 2014;46:270-8.

5 Huang S, van der Vossen EA, Kuang H, Vleeshouwers VGAA, Zhang N, Borm TJ, et al. Comparative genomics enabled the isolation of the R3a late blight resistance gene in potato. Plant J. 2005;42:251-61.

6 Simao FA, Waterhouse RM, Ioannidis P, Kriventseva EV, Zdobnov EM. BUSCO: assessing genome assembly and annotation completeness with single-copy orthologs. Bioinformatics. 2015;31:3210-2.

7 Zerbino DR, Birney E. Velvet: algorithms for *de novo* short read assembly using de Bruijn graphs. Genome Res. 2008;18:821-9.

8 Schulz MH, Zerbino DR, Vingron M, Birney E. Oases: robust *de novo* RNA-seq assembly across the dynamic range of expression levels. Bioinformatics. 2012;28:1086-92.

9 McDowall J, Hunter S. InterPro protein classification. Methods Mol Biol. 2011;694:37-47.

10 Goff SA, Ricke D, Lan TH, Presting G, Wang R, Dunn M, et al. A draft sequence of the rice genome (*Oryza sativa* L. ssp. *japonica*). Science. 2002;296:92-100.

11 The Tomato Genome Consortium. The tomato genome sequence provides insights into fleshy fruit evolution. Nature. 2012;485:635-41.

12 Potato Genome Sequencing Consortium. Genome sequence and analysis of the tuber crop potato. Nature. 2011;475:189-95.
